# Sequential gene expression analysis of cervical malignant transformation identifies RFC4 as a novel diagnostic and prognostic biomarker

**DOI:** 10.1186/s12916-022-02630-8

**Published:** 2022-11-09

**Authors:** Jianwei Zhang, Silu Meng, Xiaoyan Wang, Jun Wang, Xinran Fan, Haiying Sun, Ruoqi Ning, Bing Xiao, Xiangqin Li, Yao Jia, Dongli Kong, Ruqi Chen, Changyu Wang, Ding Ma, Shuang Li

**Affiliations:** 1 Department of Obstetrics and Gynecology, Tongji Hospital, Tongji Medical College, Huazhong University of Science and Technology, Wuhan, Hubei 430030 China; 2 Cancer Biology Research Center, Tongji Hospital, Tongji Medical College, Huazhong University of Science and Technology, Wuhan, Hubei 430030 China; 3 College of Life Sciences, University of Chinese Academy of Sciences, Beijing, 100049 China; 4 Institute of Pathology, Tongji Hospital, Tongji Medical College, Huazhong University of Science and Technology, Wuhan, Hubei 430030 China

**Keywords:** Squamous intraepithelial lesions, Cervical cancer, Molecular changes, RFC4, Biomarkers

## Abstract

**Background:**

Cervical squamous cell carcinoma (SCC) is known to arise through increasingly higher-grade squamous intraepithelial lesions (SILs) or cervical intraepithelial neoplasias (CINs). This study aimed to describe sequential molecular changes and identify biomarkers in cervical malignant transformation.

**Methods:**

Multidimensional data from five publicly available microarray and TCGA-CESC datasets were analyzed. Immunohistochemistry was carried out on 354 cervical tissues (42 normal, 62 CIN1, 26 CIN2, 47 CIN3, and 177 SCC) to determine the potential diagnostic and prognostic value of identified biomarkers.

**Results:**

We demonstrated that normal epithelium and SILs presented higher molecular homogeneity than SCC. Genes in the region (e.g., 3q, 12q13) with copy number alteration or HPV integration were more likely to lose or gain expression. The IL-17 signaling pathway was enriched throughout disease progression with downregulation of IL17C and decreased Th17 cells at late stage. Furthermore, we identified AURKA, TOP2A, RFC4, and CEP55 as potential causative genes gradually upregulated during the normal-SILs-SCC transition. For detecting high-grade SIL (HSIL), TOP2A and RFC4 showed balanced sensitivity (both 88.2%) and specificity (87.1 and 90.1%), with high AUC (0.88 and 0.89). They had equivalent diagnostic performance alone to the combination of p16^INK4a^ and Ki-67. Meanwhile, increased expression of RFC4 significantly and independently predicted favorable outcomes in multi-institutional cohorts of SCC patients.

**Conclusions:**

Our comprehensive study of gene expression profiling has identified dysregulated genes and biological processes during cervical carcinogenesis. RFC4 is proposed as a novel surrogate biomarker for determining HSIL and HSIL+, and an independent prognostic biomarker for SCC.

**Supplementary Information:**

The online version contains supplementary material available at 10.1186/s12916-022-02630-8.

## Background

Cervical cancer is the fourth most common cancer in females, with 604,127 new cases and 341,831 deaths estimated for 2020 worldwide [1]. Squamous cell carcinoma (SCC) is the predominant histological type of cervical cancer, with adenocarcinoma (AC) occurring less frequently [2]. Persistent high-risk human papillomavirus (HR-HPV) infection is associated with the development of cervical intraepithelial neoplasia (CIN), if untreated, which may progress to SCC over a period of 15 to 20 years [3]. Currently, a two-tier system of low- and high-grade squamous intraepithelial lesions (LSIL and HSIL) paralleling the terminology of the Bethesda System cytologic reports was recommended to replace the old CIN classification by World Health Organization (WHO) [4].

Cervical carcinogenesis is a complex process occurring as a consequence of multiple genomic alterations. Several expression microarray studies have been conducted investigating transcriptome changes in this process. Some research focused on specific dysregulated genes mediating the invasion of cervical cancer cells [5, 6]. Other research was designed to identify molecular changes that drive cervical cancer development [7–9]. Of note, studies based on next-generation sequencing are rare, probably due to ethical reasons and difficulties in obtaining tissue samples. Driven by the need for a comprehensive molecular characterization of the carcinogenic process, we performed a meta-analysis on publicly available gene expression profiles for an in-depth study.

This study is also motivated by the clinical desire to develop novel biomarkers of cervical carcinogenesis. On the diagnostic front, early detection of HSIL and subsequent surgical intervention are necessary to prevent further progression [10]. However, the inter- and intra-observer reproducibility of SIL grade evaluation is often poor among different pathologists due to mimics of HSIL (e.g., atrophy, LSIL, and therapy changes) [11–13]. In routine pathology practice, p16^INK4A^ and Ki-67 are the most commonly used biomarkers of HR-HPV infection and cell proliferation, respectively. It has been demonstrated that p16^INK4a^ can distinguish HSIL from its mimics and improve the diagnostic consistency of precancerous lesions among pathologists [14, 15]. Nonetheless, p16^INK4a^ has a certain positive rate in normal cervical tissue, cervicitis, and LSIL, which limits specificity for detecting HSIL [16–18]. On the prognostic front, although the incidence and mortality of cervical cancer are decreasing due to increased global vaccination and screening coverage, clinical outcomes of patients with advanced-stage or recurrence disease are still bleak and difficult to predict [19]. Driven by the need for effective biomarkers to improve the diagnosis of HSIL and the prognosis of SCC, we specifically focused on screening persistently altered genes involved in carcinogenesis.

## Methods

### Study design

**Fig. 1:**
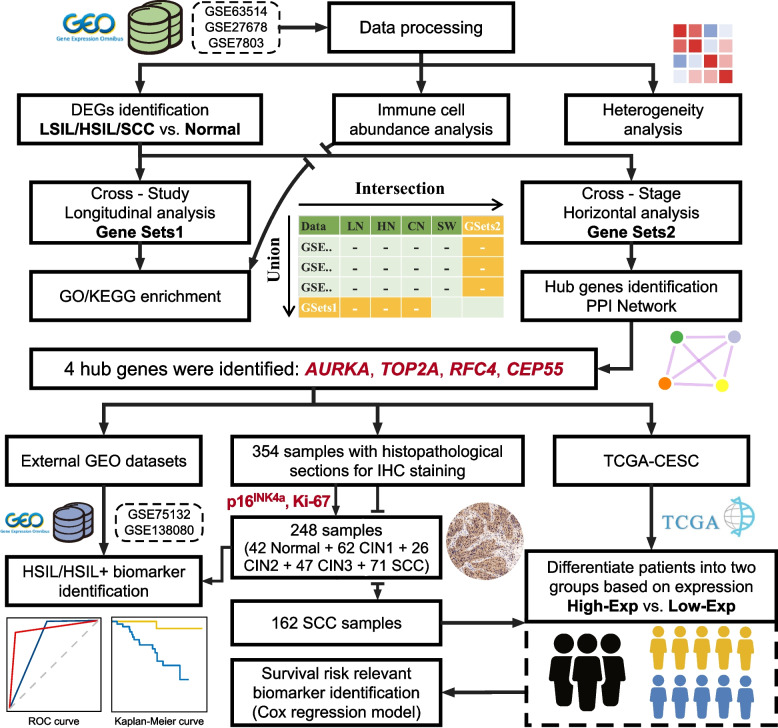
Schematic workflow of the present study. TCGA-CESC, the cancer genome atlas-cervical squamous cell carcinoma and endocervical adenocarcinoma

The overall workflow of the present study is shown in Fig. 1. Briefly, the study was undertaken in two parts. First, an integrative bioinformatic analysis of gene expression microarray datasets was conducted to identify molecular changes and hub genes linked to SCC progression. Second, external datasets and multi-institutional cohorts were used to further validate the diagnostic and prognostic robustness of selected genes from the first step.

### Data collection and preprocessing

As of Jun 06, 2020, we performed a systematic search of the Gene Expression Omnibus (GEO) and ArrayExpress databases. The inclusion criteria were as follows: (i) the datasets of mRNA expression profile; (ii) human tissue samples containing at least three disease stages from normal, LSIL (CIN1), HSIL (CIN2-3), and SCC; (iii) at least 25 samples. Four microarray datasets were included, of which three Affymetrix-based datasets (GSE63514 [7, 20], GSE27678 [5, 21], and GSE7803 [6, 22]) were used as discovery datasets. The remaining Agilent-based dataset (GSE138080 [8, 23]) and another prospective study (GSE75132 [24, 25]) were selected as validation datasets. The characteristics of the microarray datasets are summarized in Additional file 1: Table S1. The series matrix files and detailed information of array experiments were downloaded. All gene expression data had already been normalized, and a log2-based transformation was applied if the data were not log2 transformed. Boxplots of normalized microarray data can be seen in Additional file 1: Fig. S1, which showed an essentially similar distribution of expression profiles among the samples in each dataset. Afterward, the probes were mapped to genes. Genes with multiple probes were represented by the probe with the highest mean expression level.

The clinical and molecular data (including mRNA expression and copy number) of primary cervical cancer patients were retrieved from The Cancer Genome Atlas (TCGA) database through the R package TCGAbiolinks [26]. We downloaded gene expression quantitated as fragments per kilobase of transcript per million mapped reads upper quartile (FPKM-UQ) and Masked Copy Number Segment data generated by Affymetrix SNP 6.0 array. Moreover, we also downloaded the survival information from TCGA Pan-Cancer Clinical Data Resource [27].

### Cervical tissue collection

The tissue specimens (*n* = 420) were obtained from Fanpu Biotech. Co., Ltd. (FBC; Guilin, China), Department of Gynecological Oncology of Tongji Hospital (TJH; Wuhan, China), and Outdo Biotech. Co., Ltd. (OBC; Shanghai, China) (Additional file 1: Table S2). This study was conducted in accordance with the Declaration of Helsinki and approved by the Ethics Committee and Institutional Review Board of the three institutions above. Informed consent was obtained from all participants. Hematoxylin-eosin (H&E)-stained sections were reviewed by two independent pathologists (X.Y.W. and J.W.) blinded to the original diagnoses. A unanimous or majority diagnosis defined as agreement by at least two out of three diagnoses (original and the two new) was recognized as the final “gold standard” diagnosis. Finally, a total of 354 samples met the criteria for further study (Additional file 1: Fig. S2).

### Bioinformatics analysis

Differentially expressed gene (DEG) analysis was conducted between lesions and normal tissue (LSIL/HSIL/SCC vs. Normal, respectively) using the R package limma [28], with the criteria of Benjamini-Hochberg (BH) adjusted *p*-value < 0.05 and absolute fold change > 2. “Cross-study” longitudinal analysis was performed to obtain Gene Sets1, which referred to the union of upregulated genes and the union of downregulated genes per comparison after removing DEGs exhibiting inconsistent direction of change in any two discovery datasets (Fig. 1, Additional file 1: Table S3). The significance of copy number variations (CNVs) was assessed from the segmented data using GISTIC2.0 in GenePattern [29, 30]. Gene-level copy number values and frequency of CNVs were extracted for further analysis. The OmicCircos package in R [31] was utilized to visualize the expression patterns of the Sets1 genes according to a gradient of disease severity. The R package clusterProfiler [32] was used for the enrichment of Gene Sets1 and total DEGs. Cytoband enrichment was performed using positional gene sets (C1 collection available from MSigDB) with a *q*-value < 0.05. Gene Ontology (GO) and Kyoto Encyclopedia of Genes and Genomes (KEGG) pathway enrichment analyses were carried out to determine the biological significance with a *q*-value < 0.1. Selected pathway was visualized through the R package Pathview [33]. For GO enrichment in terms of biological process, we used the simplify function of clusterProfiler to remove redundant enriched terms. Then the R package GOSemSim [34] was used to estimate the pairwise semantic similarity between simplified terms for clustering. The abundance of Th17 cells was assessed with Immune Cell Abundance Identifier (ImmuCellAI) [35].

“Cross-stage” horizontal analysis was performed to obtain Gene Sets2, which referred to the intersection of DEGs and “stepwise genes” (showed gradually increasing/decreasing expression with progression of cervical lesions) per discovery dataset (Fig. 1, Additional file 1: Table S3). The protein-protein interaction (PPI) networks for Gene Sets2 were constructed with the STRING database and visualized with Cytoscape (version 3.7.1) [36, 37]. Then, we used the plug-in cytoHubba (version 0.1) [38] to rank and explore essential nodes in the interactome network. The top 10 nodes from each of the nine ranking methods (Betweenness, Bottleneck, Closeness, Degree, EPC, DMNC, MNC, Radiality, and Stress) were collected per study, and sub-PPI networks were established based on them. Any overlap in three sub-PPI networks was regarded as the “Hub Gene” of the present study.

### Immunohistochemistry (IHC) and scoring

Firstly, the tissue sections were baked at 65 °C for 30 min and then deparaffinized in xylene and passed through graded alcohol followed by antigen retrieval with 1 mM EDTA, pH 9.0 (G1203, Servicebio, Wuhan, China) in a microwave at 50 °C for 10 min, and then 30 °C for 10 min. The sections were incubated in 3% H_2_O_2_ for 25 min to quench endogenous peroxidase activity and then washed carefully in phosphate-buffered saline (PBS, pH 7.4) three times. 3% bovine serum albumin (G5001, Servicebio, Wuhan, China) was added onto the sections to cover the tissue evenly and incubated for 30 min at room temperature. The sections were subsequently incubated with the diluted antibodies (p16^INK4a^, Ki-67, AURKA, TOP2A, RFC4, CEP55) overnight at 4 °C. The details of antibodies are summarized in Additional file 1: Table S4. After carefully rinsing the sections with PBS, the sections were treated using the Pika general antibody (G1211, Servicebio, Wuhan, China; horseradish peroxidase-conjugated rabbit/mouse antibody) for 50 min, followed by diaminobenzidine (G1211, Servicebio, Wuhan, China) to detect expression under the microscope. Finally, the sections were counterstained with hematoxylin, dehydrated, and covered.

Immunohistochemical interpretations were performed independent of the H&E diagnosis by the two pathologists mentioned above. Unqualified sections were firstly discarded, and the remaining sections were evaluated for positive or negative staining, stained cellular compartment (Additional file 1: Fig. S2). For noninvasive squamous epithelia, a summary of the immunohistochemical scoring system is given in Additional file 1: Table S5. Put simply, p16^INK4a^ immunopositivity was determined following the modified version of the criteria described by Darragh et al. [39]. The cell-layer level of Ki-67 and TOP2A expression was evaluated (parabasal layer, 0; lower third of the epithelium, 1+; lower two thirds, 2+; more than lower two thirds up to full thickness, 3+). The TOP2A scores of 2+ and 3+ were grouped as 2+. AURKA and CEP55 expressions were evaluated for the staining intensity (no staining, 0; weak, 1+; moderate, 2+; strong, 3+). RFC4 expression was evaluated based on staining intensity and distribution. For SCC, samples with > 10% positive cancer cells were considered positive for all markers.

Meanwhile, AURKA, TOP2A, and RFC4 staining in SCC were assessed using the semi-quantitative histologic score (HSCORE) system. The staining intensity (0, 1+, 2+, or 3+) of cells and percentage (0–100%) of cells at each staining intensity level were estimated. The HSCORE was assigned using the following formula: HSCORE = [1 × (% cells 1+) + 2 × (% cells 2+) + 3 × (% cells 3+)], with a ranking between 0 and 300. Only the staining intensity of CEP55 was estimated for its diffuse staining in SCC.

### Survival analysis

Three independent cohorts of SCC patients (TCGA, *n* = 252; TJH, *n* = 56; OBC, *n* =106) were included for survival analysis. The clinical and pathological characteristics of the cohorts are summarized in Additional file 1: Table S6. The analysis consists of three steps. Firstly, patients in the TCGA cohort were dichotomized according to the optimal cutoff value for FPKM-UQ of hub genes. The association of gene expression (mRNA) with clinical outcomes was evaluated. Secondly, patients in the TJH cohort were dichotomized according to the optimal cutoff value for HSCORE or staining intensity of hub genes to validate the association at the protein level. Finally, the OBC cohort was entered into the TJH cohort to construct an Extended cohort (*n* = 162) for more reliable validation of the selected gene.

Different clinical outcome endpoints, overall survival (OS), progress-free interval (PFI), and disease-free survival (DFS), were defined in three cohorts. In the TCGA cohort, OS was defined as the time from initial diagnosis until death from any cause. PFI was defined as the time from initial diagnosis until recurrence of tumor, including locoregional recurrence, distant metastasis, new primary tumor, or death with tumor [27]. In the TJH and OBC cohorts, OS was defined as the time from primary surgery or the last day of therapy if no surgery until death from any cause. DFS was defined as the time from primary surgery or the last day of therapy if no surgery until recurrence of tumor or death from any cause. Patients who did not experience the event of interest were censored at the date of the last available follow-up or 5 years (whichever came first). Survival curves were plotted using the Kaplan-Meier method and compared using the log-rank test. Multivariate Cox regression analysis was performed to determine the prognostic value of the selected gene with considering clinical factors. The R packages survival, survminer, and timeROC [40] were utilized to perform the survival analysis and visualization.

### Statistical analysis

All statistical analyses were performed in the R statistical computing environment (version 3.5.3). Either Pearson’s or Spearman’s correlation coefficients were calculated to ascertain bivariate correlations. Prior to comparison, data normality was evaluated by Shapiro-Wilk test, and homogeneity of variances was evaluated by Levene’s test. Student’s *t* test, Wilcoxon rank-sum test, one-way ANOVA test, and Kruskal-Wallis test were used for numerical variables. Chi-square test and McNemar’s test were used for independent and paired categorical variables, respectively. For analysis of the association between IHC findings of biomarkers and histological evaluation, sensitivity, specificity, positive predictive value (PPV), negative predictive value (NPV), and area under the receiver operating characteristic (ROC) curve (AUC) were calculated. Cohen’s kappa coefficient (*κ*) was calculated to determine the agreement between tested IHC markers. Survival analysis has been described above. All *p*-values were two-sided, with *p* < 0.05 indicating statistical significance unless otherwise stated.

## Results

### Cervical inter- and intralesional heterogeneity assessment

**Fig. 2:**
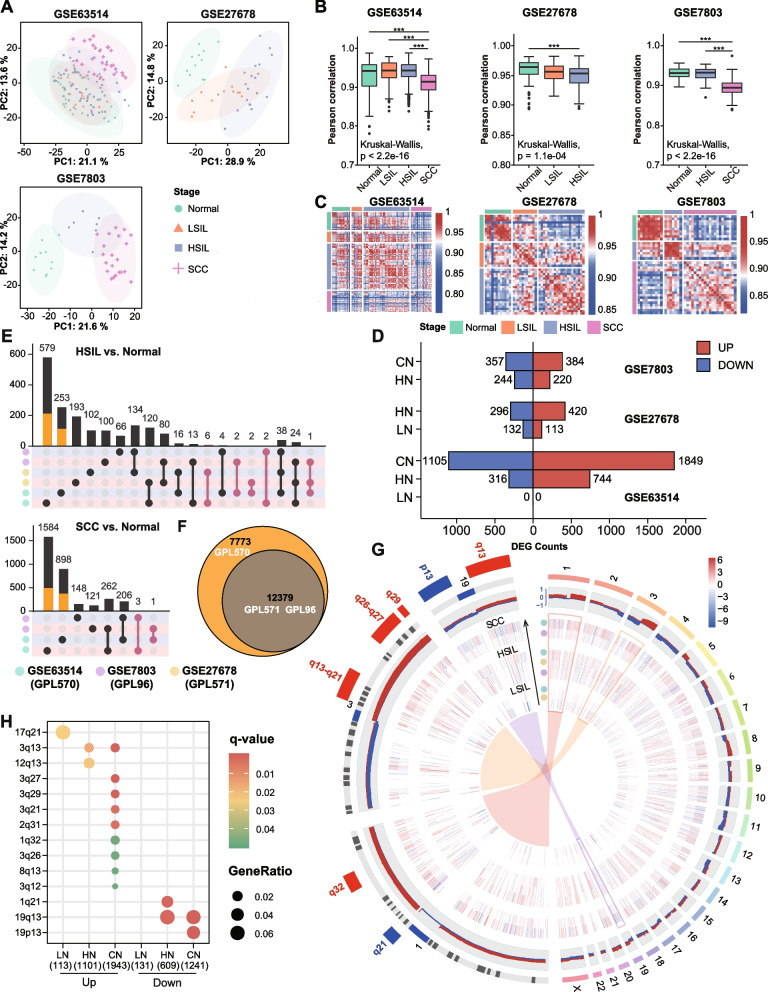
Integrated analysis. **A** Principal component analysis of the most variable 1000 genes in three datasets. The first two principal components are displayed and colored according to the disease stage. The shaded ellipses represent the 95% confidence intervals. **B** Boxplots representing Pearson correlation coefficient distribution between cases at each stage. Kruskal-Wallis test followed by pairwise Wilcoxon rank-sum test with Bonferroni correction was used to compare correlations. *** *p* < 0.001. **C** Heatmap displaying Pearson correlations between pairwise comparisons for all samples. **D** Bar plot representing the number of DEGs in each disease stage across discovery datasets. **E** UpSet diagrams showing the intersection size of DEGs in HN and CN across discovery datasets. The colors of the matrix background represent up- (red) and downregulated (blue) genes. Orange bars represent the number of DEGs explained by unique genes to GPL570. Pale violet-red dots indicate inconsistently changed genes among different studies. **F** Venn diagram showing the number of shared and unique genes annotated in GPL96, GPL570, and GPL571. **G** Circos plot illustrating landscape of chromosomal positions, expression, CNVs, and significant chromosomal bands. Autosomes 1–22 and sex chromosome X are shown in the right half of the circle. Zoomed chromosomes 1, 3, and 19 are displayed in the left half. Tracks from innermost to outermost: expression heatmap of Sets1 genes along lesion severity gradient (LN-HN-CN, Track 1-3), frequency of gains (red), and losses (blue) for regions of each chromosome from TCGA-SCC (Track 4), chromosome cytobands (Track 5), and significantly enriched cytobands in zoomed chromosomes (Track 6). **H** Dot plot showing the results of cytoband enrichment. Dot color indicates the *q*-value of the enrichment test; dot size represents the fraction of genes annotated to each cytoband. Q-value < 0.05 is considered as statistically significant

The principal component analysis (PCA) showed that normal and LSIL were not clearly discriminated in GSE63514. However, there was a distinct separation between normal and other disease stages in other datasets. The inconsistent result may be due to the different HPV statuses in normal tissue. LSIL showed higher similarity with normal epithelium with HR-HPV infection than those without HR-HPV infection (Fig. 2A, Additional file 1: Table S1). Pairwise correlation coefficients of gene expression profiles were calculated for cases at the corresponding stage to measure intralesional heterogeneity. In GSE63514, we observed significantly lower correlations for SCC as compared with HSIL, LSIL, and normal (mean Pearson’s *r*: 0.91 vs. 0.94 vs. 0.94 vs. 0.93, all *p* < 0.05), reflecting a molecular intralesional heterogeneity in SCC. Similar results were obtained from GSE7803 (Fig. 2B). Correlation heatmap of pairwise samples indicated a certain similarity between noninvasive squamous epithelium samples (Fig. 2C).

### DEG identification and cytoband enrichment analysis

To further explore the transcriptional landscape during carcinogenesis, we performed differential expression analyses between LSIL, HSIL, SCC, and normal tissues (LSIL/HSIL/SCC vs. Normal: LN, HN, CN), respectively. The number of identified DEGs was limited in LN, followed by an increase in progression (Fig. 2D). On the one hand, we observed a reasonable consensus representation of DEGs across discovery datasets in HN and CN, indicating results reliability. On the other hand, GSE63514 displayed the highest proportion of study-specific DEGs (HN: 78.5%, CN: 84.0%), about a third of which could be explained by platform-specific genes (Fig. 2 E, F). Given the aforementioned reasons, we applied “cross-study” analysis to generate the union of up- and downregulated genes separately for each comparison (including LN_UP/DN, HN_UP/DN, and CN_UP/DN, termed Gene Sets1) to combine data from multiple independent microarray datasets.

Chromosome mapping of Gene Sets1 revealed a genome-wide distribution, and regions with high-frequency chromosomal aberrations contained more specific DEGs (Fig. 2G). The deregulated genes were significantly clustered on 1q21, 1q32, 2q31, 3q, 8q13, 12q13, 17q21, 19q13, and 19p13, which correspond to previously reported HPV integration or CNV regions linked to cervical cancer. Interestingly, 1q21 and 19q13 previously described as amplification regions showed enrichment of downregulated genes. Enriched chromosome bands 3q13 and 19q13 were observed in HN and CN and only 17q21 in LN (Fig. 2H, Additional file 2: Table S7).

### Functional and signaling transition in cervical carcinogenesis

**Fig. 3:**
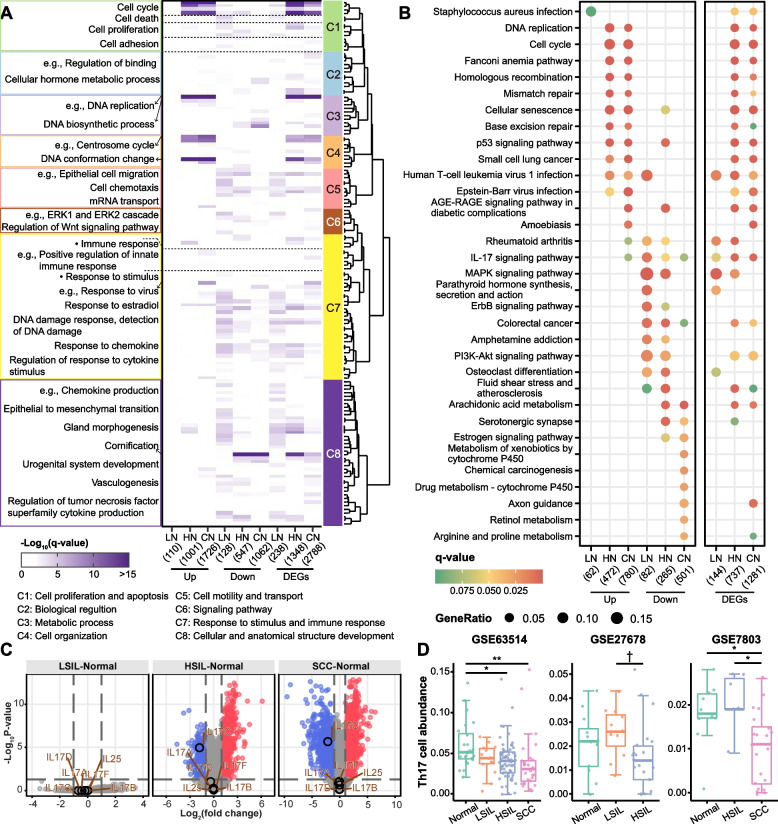
Functional enrichment and Th17 cell infiltration. **A** Heatmap showing the GO biological process terms enriched by up- and downregulated genes (Gene Sets1, 1 to 6 columns) and total DEGs (7 to 9 columns) of each comparison. The simplified GO terms significantly enriched by at least one comparison group were included and hierarchically clustered. Eight distinct clusters of GO terms that show high semantic similarity were identified. The color intensities indicate the −log_10_(*q*-value) of the enrichment test. See Additional file 2: Table S8 for the entire list of the enriched GO terms. **B** Dot plot showing the top 8 significantly enriched KEGG pathways in up- and downregulated genes (Gene Sets1, left panel) and total DEGs (right panel) of each comparison. Dot color indicates the *q*-value of the enrichment test; dot size represents the fraction of genes annotated to each pathway. The entire list of the enriched pathways and comparison can be seen in Additional file 2: Table S9 and Additional file 1: Fig. S3. **C** Volcano plots of GSE63514. Red and blue dots represent up- and downregulated genes, respectively; gray dots represent non-statistically significant genes. Vertical dashed lines indicate a 2-fold change cutoff in either direction, and horizontal dashed lines indicate an adjusted *p*-value cutoff of 0.05. IL17A through IL17F were circled and labeled with gene symbols. **D** Boxplots showing the abundance of Th17 cells changes over the disease stages. ** *p* < 0.01; * *p* < 0.05; † *p* < 0.1

GO enrichment of Gene Sets1 and total DEGs revealed a similar enrichment pattern of HN and CN. Eight distinct clusters were determined, and each was assigned a unique enrichment signature (Fig. 3A). We observed that upregulated genes of HN and CN showed a strong enrichment for cell cycle and related terms (Clusters 1, 3, 4). Intriguingly, the positive regulation of cell cycle term was enriched with LN_DN, HN_UP/DN, and CN_UP genes, indicating a dynamic regulation of cell cycle control. Consistent with dysmaturation of keratinocytes throughout disease progression, downregulated genes of HN and CN showed significant enrichment for cornification term (Cluster 8). In addition, LN_UP genes were not enriched in any term, while LN_DN genes were mainly enriched in Clusters 1, 5–8 (Fig. 3A, Additional file 2: Table S8).

KEGG enrichment of total DEGs showed a great agreement between HN and CN. While in the enrichment of downregulated genes, HN appears to be a transitional state since the enriched pathways of HN were partially overlapped with that of CN and LN (Additional file 1: Fig. S3). DNA repair pathways [homologous recombination (HR), base excision repair (BER), nucleotide excision repair (NER) and mismatch repair (MMR)], cell cycle-related pathways (cell cycle, DNA replication, and cellular senescence), and oncogenic p53 signaling pathways were enriched with upregulated genes of HN and CN (Fig. 3B, Additional file 2: Table S9). Downregulated genes of LN and HN were enriched in more cancer-related pathways (e.g., MAPK, ErbB, PI3K/AKT, TGF-β, and Wnt signaling pathways) than that of CN. Some pathways above, such as PI3K/AKT and Wnt pathways, could also be enriched in CN when considering total DEGs (Fig. 3B, Additional file 2: Table S9).

Human T-cell leukemia virus 1 (HTLV-1) infection and immune-related IL-17 signaling pathway were enriched in all disease stages among DEGs (Fig. 3B). Many DEGs of HN and CN enriched in HTLV-1 infection signaling were cell cycle-related genes, and most of which are upregulated. Ten genes in HTLV-1 infection signaling were differentially expressed in LN, and all serum response factor (SRF) pathway genes (SRF, FOS, FOSL1, EGR1, and EGR2) were involved and downregulated (Additional file 1: Fig. S4, Additional file 2: Table S9). IL17 cytokine family comprises IL17A, IL17B, IL17C, IL17D, IL17E (also known as IL25), and IL17F. Among these cytokine genes, only IL17C was founded to be differentially expressed (downregulated) in HN and CN (Fig. 3C, Additional file 2: Table S9). IL17C was produced primarily by keratinocytes, gradually replaced by abnormal epithelial cells in malignant transformation. Although Th17-associated cytokine genes (IL17A and IL17F) were not affected, we still observed that Th17 cells showed significantly decreased abundance at the late stage of cancer progression (Fig. 3D), which is inconsistent with previous findings [41].

### Identification of the potential genes associated with carcinogenesis

**Fig. 4:**
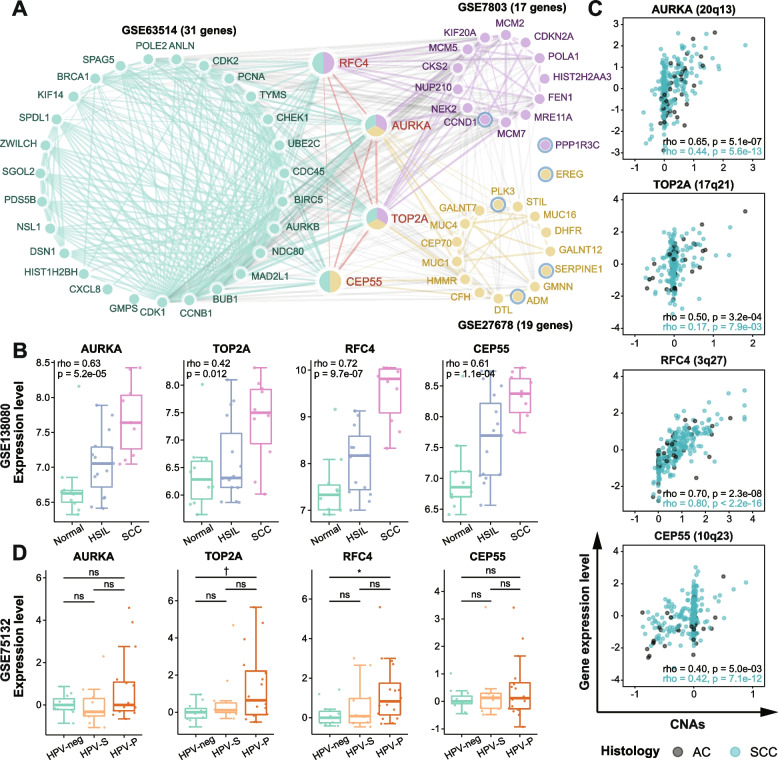
Hub genes identification and validation. **A** PPI network of important DEGs selected by Cytohubba. The nodes with white and blue rings denote progressively up- and downregulated genes with the development of cervical lesions (Spearman, *p* < 0.05). Edge thickness is proportional to the interaction score. **B** Boxplots showing the correlations between hub gene expression and severity of cervical lesion in GSE138080, with Spearman’s rho and *p*-values presented in the upper left corner. **C** Scatter plots showing the correlations between hub gene expression (*Z* score-transformed log_2_ (FPKM-UQ+1) values) and CNAs in TCGA-CESC dataset, with Spearman’s rho and *p*-values presented in the lower right corner. Adenocarcinoma (AC) samples are shown in black and squamous cell carcinoma (SCC) samples are shown in blue. **D** Boxplots showing hub gene expression in HPV-neg, HPV-S (HPV16 persistent infection without progression), and HPV-P (HPV16 persistent infection with progression) women from GSE75132. Statistical comparisons were performed using Wilcoxon rank-sum test. * 0.01 < *p* < 0.05; † *p* < 0.1; ns, *p* ≥ 0.1

To identify pivotal DEGs and provide clues for early diagnosis and intervention of precancerous lesions, we applied “cross-stage” analysis to generate the intersection of DEGs and “stepwise genes” for each discovery dataset (termed Gene Sets2). Through PPI network analysis of Sets2 genes, four genes (AURKA, TOP2A, CEP55, and RFC4) occurring in at least two datasets were considered as “Hub Genes” (Fig. 4A, Additional file 1: Fig. S5). Hub gene expression increased significantly during progression from normal to SCC (all *p* < 0.05, Additional file 1: Fig. S6), which were also validated in GSE138080 (all *p* < 0.05, Fig. 4B). Furthermore, these genes had been proved to be involved in the malignant transformation of several different types of tumors, like Barrett’s adenocarcinoma, colorectal carcinoma, head and neck squamous cell carcinoma (HNSCC), etc. As expected, they tended to display a linear expression pattern with tumor progression (Additional file 1: Table S10).

The observed disease stage-dependent expression changes could be induced by multi-factors such as genetic and epigenetic alterations, small RNAs, and HPV integrations. This study assessed associations between hub gene expression level and corresponding copy number alterations (CNAs) in AC and SCC. We found a strong positive correlation between expression level and CNAs for RFC4 (Spearman’s rho: 0.70 and 0.80, all *p* < 0.05), and a moderate correlation for AURKA (Spearman’s rho: 0.65 and 0.44, all *p* < 0.05) and CEP55 (Spearman’s rho: 0.40 and 0.42, all *p* < 0.05) in AC and SCC (Fig. 4C). Interestingly, CNAs of TOP2A have a tumor subtype-specific role in contributing to gene expression variability. AC shows a tighter correlation than SCC between those two parameters (Spearman’s rho: 0.50 vs. 0.17, Fig. 4C).

Moreover, we examined whether the expression levels of hub genes were predictive for developing high-grade cervical lesions in a prospective cohort study (GSE75132). The study enrolled HPV-negative and persistently HPV16-infected women. HPV-infected women were divided into progressor (HPV-P) and sustainer (HPV-S) groups according to whether they progressed to CIN3+ or not during follow-up (Additional file 1: Table S1). We noted a trend toward a higher median expression level of RFC4 and TOP2A in HPV-P women than in HPV-S and HPV-negative women. Their expression differences between HPV-P and HPV-negative women were significant or marginally significant, respectively (*p* = 0.034 for RFC4 and *p* = 0.08 for TOP2A, Fig. 4D).

### Diagnostic assessment of hub genes for HSIL and HSIL+

**Fig. 5:**
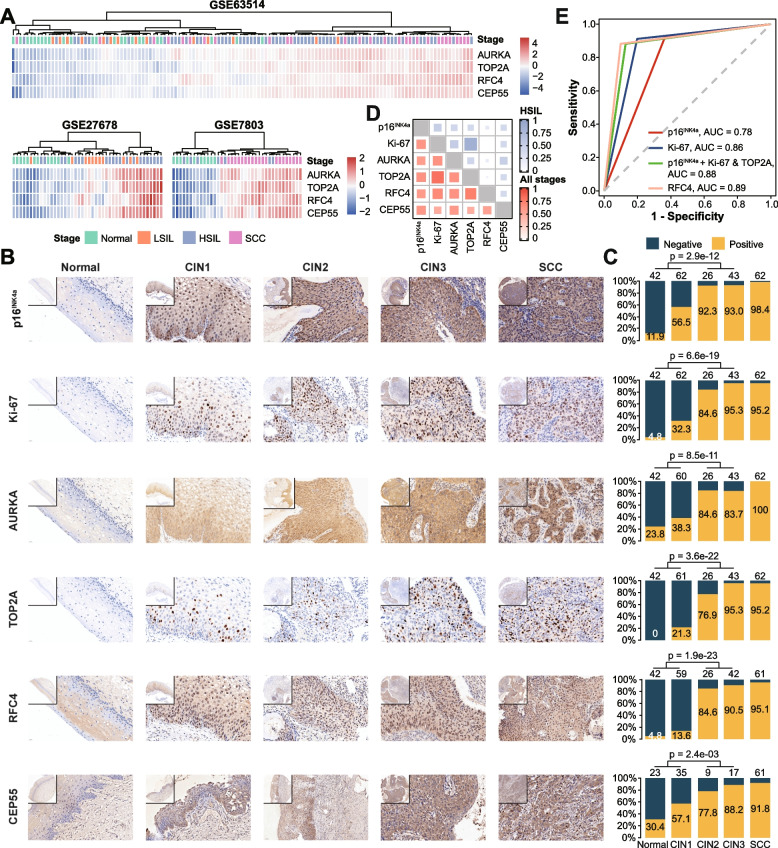
p16^INK4A^, Ki-67, AURKA, TOP2A, RFC4, and CEP55 immunohistochemistry and potential diagnostic utility. **A** Heatmap illustrating the hierarchical clustering of samples (columns) from discovery datasets based on the scaled expression of hub genes (rows). Blue to red spectrum color gradient indicates low to high expression level. **B** Representative IHC staining images of tested markers in normal, CIN1-3, SCC tissues. Original magnification ×400. Inserts, original magnification ×100. **C** Stacked bar plots showing the fraction of positively (orange) and negatively staining (blue) samples in each disease stage, with positive rates presented. The *p*-values indicate the difference in the distribution of positive and negative samples between normal/LSIL and HSIL (chi-square test). The numbers above each bar refer to the number of samples in each stage. **D** Heatmap of kappa statistics for tested IHC markers in HSIL (blue) and all stages (red), related to Additional file 1: Fig. S13. **E** ROC curves for comparison of single and combined biomarkers in HSIL diagnosis and associated AUC values were shown (also see Table 1). ROC, receiver operating characteristic; AUC, area under the ROC curve

Hierarchical clustering of hub genes revealed a good separation between normal/LSIL and HSIL+ (HSIL and SCC) (Fig. 5A). Thus, we performed IHC for p16^INK4a^, Ki-67, and four hub genes to validate the identified transcriptomic changes at the protein level and explore their clinical utility in diagnosing HSIL and HSIL+. Corresponding to changes in mRNA, the protein expression of all tested markers gradually increased from normal to CIN3 (Fig. 5B, Additional file 1: Table S11). Because of different scoring systems, we could not compare marker expression between normal/CINs and SCC by IHC score. However, the neoplastic epithelial cells of SCC showed stronger staining (increased positive intensity and more diffuse expression) than those of CIN3 according to subjective visual estimation (Fig. 5B).

Cellular markers were classified into three categories based on their biological function, including cell cycle regulation marker (p16^INK4a^, AURKA, RFC4), cell division marker (CEP55), and proliferation marker (Ki-67 and TOP2A). Then we developed an individual scoring system to evaluate staining intensity and extent (Additional file 1: Fig. S7, Table S5). The positive rates of all markers significantly increased with the severity of cervical lesion, especially in HSIL and SCC (Fig. 5C). For p16^INK4a^ and Ki-67, our results were in good correspondence with previous studies, which improved confidence in the reliability of IHC and comparability of evaluation. Meanwhile, the previously reported frequency of positive TOP2A and ProExC (MCM2 and TOP2A) in cervical lesions were also collected (Additional file 1: Table S12). The concordance analysis demonstrated a strong agreement of 90.2% (*κ* = 0.8) in the whole stages and 97.1% (*κ* = 0.84) in HSIL for Ki-67 and TOP2A (Fig. 5D, Additional file 1: Table S13).

For detecting HSIL, sensitivities, specificities, PPVs, NPVs, and AUCs of six markers are shown in Additional file 1: Table S14. Of note, CEP55 and AURKA with high sensitivity and low specificity were excluded for further comparative analysis due to their cytoplasmic staining, which made assessment challenging and interpretation difficult. To statistically compare the diagnostic utility of the remaining markers, sections with these markers evaluated simultaneously were analyzed (Additional file 1: Table S15, Table 1). Data showed the highest sensitivity of p16^INK4a^, but the specificity and PPV were low. In contrast to p16^INK4a^, Ki-67 and TOP2A provided a nearly equivalent sensitivity (92.6% vs. 91.2% vs. 88.2%, all *p* > 0.05) and a higher specificity (63.4% vs. 80.2% vs. 87.1%, all *p* < 0.05). The inclusion of Ki-67 could improve specificity (87.1% vs. 63.4%, *p* < 0.05), PPV (82.2% vs. 63%), and accuracy (AUC, 0.88 vs. 0.78) of p16^INK4a^ (Fig. 5E), at the little expense of sensitivity (92.6 to 88.2%) and NPV (92.8 to 91.7%). TOP2A had the same performance as p16^INK4a^ and Ki-67 combined. Additionally, RFC4 not only had same sensitivity (88.2% vs. 88.2%, *p* = 1) and relatively higher specificity (90.1 vs. 87.1%, *p* = 0.58), but also higher accuracy (AUC, 0.89 vs. 0.88; Fig. 5E) than the combination of p16^INK4a^ and Ki-67. Meanwhile, the diagnostic efficacy of RFC4 and TOP2A improved for detecting HSIL+ as the AUC reached 0.91 and 0.89, respectively (Table 1). These results suggest RFC4 and TOP2A alone could be complementary surrogate markers to p16^INK4a^ and Ki-67 for detecting HSIL and HSIL+.

**Table 1:** Comparison of diagnostic performance of IHC biomarkers for detecting HSIL and HSIL+

Biomarker	Sensitivity (95% CI), %	***p***-value^a^	Specificity (95% CI), %	***p***-value^a^	PPV (95% CI), %	NPV (95% CI), %	AUC (95% CI)
**HSIL**							
p16^INK4a^	92.6 (83.7–97.6)	Ref/0.248	63.4 (53.2–72.7)	Ref/<0.001	63.0 (52.8–72.4)	92.8 (83.9–97.6)	0.78 (0.72–0.84)
Ki-67	91.2 (81.8–96.7)	1.000/0.480	80.2 (71.1–87.5)	0.004/0.023	75.6 (64.9–84.4)	93.1 (85.6–97.4)	0.86 (0.81–0.91)
p16^INK4a^ + Ki-67^b^	88.2 (78.1–94.8)	0.248/Ref	87.1 (79.0–93.0)	<0.001/Ref	82.2 (71.5–90.2)	91.7 (84.2–96.3)	0.88 (0.83–0.93)
TOP2A	88.2 (78.1–94.8)	0.450/1.000	87.1 (79.0–93.0)	<0.001/1.000	82.2 (71.5–90.2)	91.7 (84.2–96.3)	0.88 (0.83–0.93)
RFC4	88.2 (78.1–94.8)	0.505/1.000	90.1 (82.5–95.1)	<0.001/0.579	85.7 (75.3–92.9)	91.9 (84.7–96.4)	0.89 (0.84–0.94)
**HSIL+**							
p16^INK4a^	95.3 (90.2–98.3)	Ref/0.041	63.4 (53.2–72.7)	Ref/<0.001	76.9 (69.6–83.2)	91.4 (82.3–96.8)	0.79 (0.74–0.84)
Ki-67	93.0 (87.2–96.8)	0.505/0.248	80.2 (71.1–87.5)	0.004/0.023	85.7 (78.8–91.1)	90.0 (81.9–95.3)	0.87 (0.82–0.91)
p16^INK4a^ + Ki-67^b^	90.7 (84.3–95.1)	0.041/Ref	87.1 (79.0–93.0)	<0.001/Ref	90.0 (83.5–94.6)	88.0 (80.0–93.6)	0.89 (0.85–0.93)
TOP2A	91.5 (85.3–95.7)	0.228/1.000	87.1 (79.0–93.0)	<0.001/1.000	90.1 (83.6–94.6)	88.9 (81.0–94.3)	0.89 (0.85–0.93)
RFC4	91.5 (85.3–95.7)	0.267/1.000	90.1 (82.5–95.1)	<0.001/0.579	92.2 (86.1–96.2)	89.2 (81.5–94.5)	0.91 (0.87–0.95)

^a^Exact McNemar’s test comparing to p16^INK4a^ and p16^INK4a^ + Ki-67

^b^Both p16^INK4a^ and Ki-67 positive

Moreover, serial and parallel interpretation of any marker pairs were compared to TOP2A and RFC4 alone (Additional file 1: Table S16). The combination of TOP2A and RFC4 in parallel interpretation with the highest accuracy (AUC, 0.90) showed significantly higher sensitivity (97.1 vs. 88.2%, *p* < 0.05) but lower specificity (82.2 vs. 90.1%, *p* < 0.05) compared with RFC4 for detecting HSIL (Additional file 1: Table S16a). None of the combinations presented certain advantages over RFC4 for detecting HSIL+ (Additional file 1: Table S16b).

### Prognostic assessment of hub genes for SCC

To further investigate the clinical impact of hub genes in SCC progression, we examined the correlation between their expression and disease severity in SCC patients from the TCGA cohort. The analysis revealed that increased AURKA mRNA expression was significantly or marginal significantly associated with advanced FIGO stage (*p* = 0.039), higher histological grade (*p* = 0.01), and lymph nodes metastasis (*p* = 0.088; Additional file 1: Fig. S8).

**Fig. 6:**
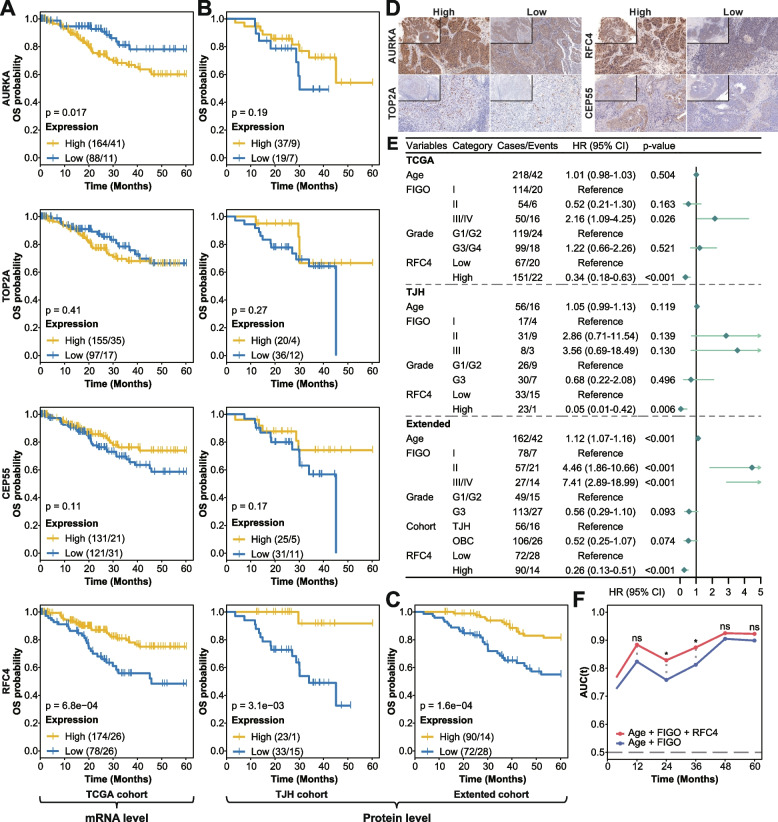
Univariate and multivariate survival analysis for OS in SCC patients, related to Additional file 1: Fig. S9. Five-year Kaplan-Meier curves for OS in SCC patients stratified by the hub gene expression (mRNA and protein) from **A** TCGA, **B** TJH, and **C** Extended cohorts. The number of cases and events are shown in the plots. The *p*-values were calculated with the log-rank test. The optimal cutoff values for HSCORE or staining intensity determined by the *surv_cutpoint* function from the survminer package were 130 for AURKA, 1 (staining intensity) for CEP55, 45 for TOP2A, and 100/105 (TJH/Extended) for RFC4. **D** Representative IHC staining images of high and low AURKA, TOP2A, RFC4, and CEP55 expression in SCC. Original magnification ×200. Inserts, original magnification ×100. **E** Forest plot of multivariate Cox regression with clinical features and RFC4 expression taken into account in three cohorts. The main effects are shown as hazard ratios with 95% confidence intervals. **F** Time-dependent AUC for combined RFC4 expression and clinical covariate model (red) and clinical covariate-only model (blue). The significant difference in the AUC was estimated at 1, 2, 3, 4, and 5 years, and adjusted *p*-values were calculated. HR, hazard ratio; CI, confidence interval. * 0.01 < *p* < 0.05; ns, *p* ≥ 0.1

Next, we evaluated the effect of hub gene expression alterations on prognosis in three independent cohorts of SCC patients (see the “Methods” section). In the TCGA cohort (*n* = 252), high AURKA mRNA expression inversely correlated with OS (log-rank, *p* = 0.017; Fig. 6A). While higher RFC4 mRNA expression was significantly associated with better OS and PFI (log-rank, *p* = 6.8e−04 and *p* = 7.8e−03, respectively; Fig. 6A, Additional file 1: Fig. S9A). To validate the finding at the protein level, we performed immunostaining for these genes on SCC tissues from the TJH cohort (*n* = 56). Overexpression of RFC4 protein showed significant associations with increased OS and DFS (log-rank, *p* = 3.1e−03 and *p* = 1.5e−03, respectively; Fig. 6B, Additional file 1: Fig. S9B). In addition, higher TOP2A protein expression significantly correlated with better DFS (log-rank, *p* = 0.019; Additional file 1: Fig. S9B), which was not found at the mRNA level. Although not statistically significant, there was a tendency for higher CEP55 expression associated with increased PFI (log-rank, *p* = 0.1) in the TCGA cohort and increased DFS (log-rank, *p* = 0.072) in the TJH cohort (Fig. 6B, Additional file 1: Fig. S9B). Due to limited samples of the TJH cohort, we further investigated the RFC4 prognostic value in the combined TJH and OBC cohort (Extended cohort, *n* = 162; Additional file 1: Fig. S10). The effect of RFC4 protein expression on OS and DFS, as expected, remained significant (log-rank, *p* = 1.6e−04 and *p* = 2.2e−04, respectively; Fig. 6C, Additional file 1: Fig. S9C). The representative IHC staining images of hub genes in SCC are shown in Fig. 6D.

Notably, multivariate Cox regression analysis revealed that RFC4 expression (mRNA and protein), after adjustment for age, FIGO stage, grade, and cohort, emerged as an independent predictor of clinical outcomes (OS, PFI, and DFS) for SCC patients in three cohorts (Fig. 6E, Additional file 1: Fig. S9D). The time-dependent AUC showed that the addition of RFC4 expression into the Cox proportional hazards model significantly increased the prognostic efficacy for 2- and 3-year OS (all *p* < 0.05; Fig. 6F), for 1-, 2-, and 3-year DFS (all *p* < 0.05; Additional file 1: Fig. S9E).

## Discussion

This meta-analysis based on previous studies comprehensively characterizes the transcriptomic profiles of cervical carcinogenesis and identifies four key genes (AURKA, TOP2A, RFC4, CEP55) associated with the initiation and progression of SCC. Then, we carefully assess their diagnostic performance in HSIL/HSIL+ and prognostic performance in SCC. To the best of our knowledge, our study is the first to evaluate and validate the diagnostic and prognostic value of RFC4 in cervical lesions.

We found that the transcriptomes of normal epithelium and SILs were homogenous. However, the increased heterogeneity was observed upon progression to SCC. A study of the transcriptomic landscape of hepatocarcinogenesis presented homogeneity in dysplastic lesions and early carcinoma but heterogeneity in advanced liver cancer, somewhat similar to our results [42]. Due to the lack of FIGO staging and histological grading data, whether there is heterogeneity between early SCC and preinvasive or late SCC was unknown in our discovery datasets. In the PCA of GSE63514, HSIL was partially overlapped with normal/LSIL and SCC. Moreover, HSIL showed higher heterogeneity than normal in GSE27678. Akin to genetic alteration, we believe that some dysregulated genes common to SCC but only changed in a part of HSIL contributed to the potential heterogeneity of HSIL [43, 44].

Compared to enrichment with total DEGs, separate enrichment with up- and downregulated genes could detect more pathways associated with the phenotypic difference [45]. We used both strategies in this study. Although separate analysis consistently detected more terms and pathways, some pathways (e.g., Wnt signaling pathway in LN_DN and CN_DEG) enriched in different disease stages by two strategies respectively should not be ignored. Cell cycle, DNA repair, and oncogenic p53 pathways were activated in HSIL and SCC. The close association between these pathways and HPV has been evidenced. HR-HPV E6 and E7 oncoproteins interfere with p53 and pRB, leading to cell cycle disturbances and promoting DNA damage response (DDR) that has a known central role in cervical carcinogenesis [3, 46, 47]. Furthermore, we found inhibition of TGF-β and Hippo signaling pathways in LSIL, consistent with their tumor-suppressive properties in the early stage of carcinogenesis [48, 49]. Interestingly, the HTLV-1 infection and IL-17 signaling pathways were enriched in all disease stages. The deregulation of cell cycle is a common feature in cancer cells and HTLV-1-infected cells, which is why we believe that the HTLV-1 infection pathway was enriched in HN and CN [50, 51]. The HTLV-1 Tax oncoprotein interacts with SRF to activate the transcription of immediate early genes (FOS, FOSL1, EGR1, and EGR2) [52, 53]. However, these genes were downregulated in LN. The association between HPV and SRF in early stage of cervical carcinogenesis might be worth investigating. Of IL-17 cytokine genes, IL17C showed significantly lower expression in HSIL and SCC when compared to normal control. IL17C is an epithelial cell-derived cytokine that regulates innate epithelial immune responses [54], and its response to HPV infection has not been explicitly investigated. A previous study had reported that increased Th17 cells were associated with progression of SCC [41], which was inconsistent with our results. However, there were studies reporting that lymph nodes of premalignant lesion-bearing mouse contained more Th17 cells than HNSCC-bearing mouse lymph nodes [55, 56]. Reduced IL23 production and increased TGF-β production by HNSCC may lead to the decrease in Th17 by redirecting the immune phenotype toward Treg [56].

We found four hub genes through network analysis. Using IHC, the gradually increasing expression of hub genes along with the severity of lesions was validated. Notably, CEP55 was initially reported to be associated with the course of cervical lesions. The staining pattern of TOP2A was similar to that of Ki-67, and concordance between them was substantial. While a study comparing ProExC and Ki-67 expression in 197 cervical biopsies reported that 35% of cases showed discordant staining [57]. We then compared the diagnostic performance of p16^INK4a^, Ki-67, TOP2A, and RFC4 alone or in combination to detect HSIL/HSIL+. Among the four markers, p16^INK4a^ routinely used in clinical practice showed the highest sensitivity but moderate specificity. Similar to previous reports, the combination of p16^INK4a^ and Ki-67 in serial interpretation could improve specificity and accuracy for detecting HSIL [58]. RFC4 and TOP2A alone provided similar diagnostic performance to the combination of p16^INK4a^ and Ki-67. Parallel interpretation of TOP2A and RFC4 produced the highest AUC, and parallel interpretation of Ki-67 and RFC4 produced the highest sensitivity and NPV for detecting HSIL. Importantly, RFC4 and TOP2A have additional advantages. The expression of RFC4 from 3q26 exhibited a high correlation with copy number gain, and 3q gain as a potential marker in the diagnosis of HSIL is frequently found in cervical cancer and its precancerous lesions [59, 60]. For TOP2A, its exclusive and clear nuclear staining is an advantage over nuclear and cytoplasmic staining of RFC4 and p16^INK4a^. Moreover, Shi et al. reported that TOP2A is more sensitive and specific than ProEXC for detecting HSIL [61]. Considering cost-effectiveness, a single biomarker with balanced sensitivity, specificity, and high accuracy is recommended. When meeting patients with suspected HSIL, we can choose parallel interpretation of Ki67/TOP2A and RFC4 with high sensitivity and NPV to safely exclude lesions.

Furthermore, we explored the clinical and prognostic significance of identified genes in SCC. Compared with a continuous increase of hub gene expression in normal to SILs to SCC transitions, only AURKA mRNA expression significantly increased with advancing FIGO stage, increasing tumor differentiation and aggressiveness in SCC, as indicated by the poor OS. This is consistent with the findings observed previously [62], though the prognostic interest of AURKA could not be validated in the TJH cohort. A previous study demonstrated that high CEP55 protein expression correlates with better OS and recurrence-free survival (RFS) in SCC [63]. We found this trend in our research but not statistically significant. Several studies based on TCGA-CESC data have reported the relationship between TOP2A and RFC4 mRNA expression and the prognosis of cervical cancer [64, 65]. However, there was no other evidence to support their relationship, let alone the confirmation at the protein level. Here, we demonstrate for the first time that increased RFC4 and TOP2A protein expression correlates with a favorable outcome in patients with SCC, and RFC4 is an independent prognostic marker for SCC. Furthermore, preliminary investigations have also demonstrated the role of RFC4 in predicting the outcome of other neoplasia, such as non-small cell lung carcinoma, colorectal cancer, and breast tumor [66–68].

Of course, our research has some limitations. Firstly, diagnosis error cannot be excluded entirely because the histopathologic diagnosis of CIN is subject to substantial rates of discordance among pathologists. Due to the majority diagnosis from three expert gynecologic pathologists and the large sample size in our study, we considered this diagnosis bias only to influence results to a minor degree. Secondly, we focus on RFC4 dynamic expression and clinical application here, which could not clarify the cause-and-effect relationship between RFC4 overexpression and disease progression. Our laboratory has ongoing experimental studies of RFC4 in papillomavirus oncogenic cell transformation.

## Conclusions

Collectively, our study has characterized the changes in gene expression and biological functions during cervical carcinogenesis, which contribute toward a better understanding of molecular mechanisms associated with disease progression. Furthermore, we have found that RFC4 and TOP2A alone could serve as potential surrogate markers for determining HSIL and HSIL+. Their potential clinical application in cytological specimens was foreseen. Finally, RFC4 was also confirmed as an independent prognostic biomarker for SCC, implicating its therapeutic targeting for the treatment of SCC.

## Supplementary Information

**Additional file 1: Figure S1.** The quality control of selected datasets. **Figure S2.** The flow of sample selection and immunohistochemistry (IHC), related to Table S2. **Figure S3.** Pathway enrichment and comparison. **Figure S4.** Human T-cell leukemia virus 1 (HTLV-1) infection signaling pathway map. **Figure S5.** Differential expression of total genes in each comparison group of the discovery datasets. **Figure S6.** Correlation between hub gene expression and severity of cervical lesion in the discovery datasets. **Figure S7.** Schematic diagram of immune scoring criteria, related to Table S5. **Figure S8.** Assessment of the relationship between hub gene expression and clinical parameters in SCC patients from the TCGA cohort. **Figure S9.** Univariate and multivariate survival analysis for PFI/DFS in SCC patients, related to Fig. 6. **Figure S10.** The illustration of the tissue microarray (HUteS154Su01; TMA) from Outdo Biotech. Co., Ltd. **Table S1.** Microarray datasets analyzed in this study. **Table S2.** Histology information of all tissue specimens, related to Figure S2. **Table S3.** Summary of DEGs and Gene Sets1&2. **Table S4.** Antibodies used for immunohistochemical staining. **Table S5.** Immunohistochemical scoring system for noninvasive squamous epithelia. **Table S6.** Clinical characteristics of the collected cohorts for survival analysis. **Table S10.** Summary of the hub gene expression related to cancer progression. **Table S11.** The summary of immunohistochemical scoring results. **Table S12.** Characteristics of studies assessing p16^INK4a^, Ki-67, TOP2A and ProExC immunohistochemically. **Table S13.** Concordance analysis between IHC biomarkers. **Table S14.** Sensitivity, specificity, PPV, NPV, and AUC of six IHC biomarkers for detecting HSIL and HSIL+. **Table S15.** Comparison of positive rates of single and combined IHC biomarkers in different cervical lesions (based on sections with p16^INK4a^, Ki-67, TOP2A and RFC4 evaluated simultaneously). **Table S16.** Diagnostic performance of serial and parallel interpretation of IHC biomarker combinations for detecting HSIL and HSIL+ compared to TOP2A and RFC4 alone.

**Additional file 2: Table S7.** Enrichment of chromosomal region. **Table S8.** Simplified results of GO enrichment. **Table S9.** KEGG enrichment results.

## Data Availability

Publicly available datasets were analyzed in this study. These can be found in The Cancer Genome Atlas (https://portal.gdc.cancer.gov/) and the NCBI Gene Expression Omnibus (http://www.ncbi.nlm.nih.gov/geo/). The tissue microarrays were obtained from Fanpu Biotech. Co., Ltd. (Guilin, China; Product code: CIN1021 and CIN1022) and Outdo Biotech. Co., Ltd. (Shanghai, China; Product Code: HUteS154Su01). The raw experimental data and analysis codes supporting the conclusions of this article will be made available by the corresponding author.

## Abbreviations

AC: Adenocarcinoma
AUC: Area under the ROC curve
BER: Base excision repair
BH: Benjamini-Hochberg
CESC: Cervical squamous cell carcinoma and endocervical adenocarcinoma
CIN: Cervical intraepithelial neoplasia
CNA: Copy number alteration
CNV: Copy number variation
DDR: DNA damage response
DEG: Differentially expressed gene
DFS: Disease-free survival
FBC: Fanpu Biotech. Co., Ltd.
FPKM-UQ: Fragments per kilobase of transcript per million mapped reads upper quartile
GEO: Gene Expression Omnibus
GO: Gene Ontology
H&E: Hematoxylin-eosin
HR: Homologous recombination
HR-HPV: High-risk human papillomavirus
HSCORE: Histologic score
HSIL: High-grade squamous intraepithelial lesion
HTLV-1: Human T-cell leukemia virus 1
IHC: Immunohistochemistry
KEGG: Kyoto Encyclopedia of Genes and Genomes
LSIL: Low-grade squamous intraepithelial lesion
MMR: Mismatch repair
NER: Nucleotide excision repair
NPV: Negative predictive value
OBC: Outdo Biotech. Co., Ltd.
OS: Overall survival
PBS: Phosphate-buffered saline
PCA: Principal component analysis
PFI: Progress-free interval
PPI: Protein-protein interaction
PPV: Positive predictive value
RFS: Recurrence-free survival
ROC: Receiver operating characteristic
SCC: Cervical squamous cell carcinoma
SRF: Serum response factor
SIL: Squamous intraepithelial lesion
TCGA: The Cancer Genome Atlas
TJH: Tongji Hospital
WHO: World Health Organization
