# Supplementary material for: Sequential gene expression analysis of cervical malignant transformation identifies RFC4 as a novel diagnostic and prognostic biomarker
Source: BMC Med. 2022 Nov 9;20:437. doi: 10.1186/s12916-022-02630-8 (PMC9648022; doi:10.1186/s12916-022-02630-8)
Supplement: Supplementary file 1 — Additional file 1: Figure S1. The quality control of selected datasets. Figure S2. The flow of sample selection and immunohistochemistry (IHC), related to Table S2. Figure S3. Pathway enrichment and comparison. Figure S4. Human T-cell leukemia virus 1 (HTLV-1) infection signaling pathway map. Figure S5. Differential expression of total genes in each comparison group of the discovery datasets. Figure S6. Correlation between hub gene expression and severity of cervical lesion in the discovery datasets. Figure S7. Schematic diagram of immune scoring criteria, related to Table S5. Figure S8. Assessment of the relationship between hub gene expression and clinical parameters in SCC patients from the TCGA cohort. Figure S9. Univariate and multivariate survival analysis for PFI/DFS in SCC patients, related to Fig. 6. Figure S10. The illustration of the tissue microarray (HUteS154Su01; TMA) from Outdo Biotech. Co., Ltd. Table S1. Microarray datasets analyzed in this study. Table S2. Histology information of all tissue specimens, related to Figure S2. Table S3. Summary of DEGs and Gene Sets1&2. Table S4. Antibodies used for immunohistochemical staining. Table S5. Immunohistochemical scoring system for noninvasive squamous epithelia. Table S6. Clinical characteristics of the collected cohorts for survival analysis. Table S10. Summary of the hub gene expression related to cancer progression. Table S11. The summary of immunohistochemical scoring results. Table S12. Characteristics of studies assessing p16INK4a, Ki-67, TOP2A and ProExC immunohistochemically. Table S13. Concordance analysis between IHC biomarkers. Table S14. Sensitivity, specificity, PPV, NPV, and AUC of six IHC biomarkers for detecting HSIL and HSIL+. Table S15. Comparison of positive rates of single and combined IHC biomarkers in different cervical lesions (based on sections with p16INK4a, Ki-67, TOP2A and RFC4 evaluated simultaneously). Table S16. Diagnostic performance of serial and parallel interpretation of [file 12916_2022_2630_MOESM1_ESM.pdf]

Additional file 1

Sequential gene expression analysis of cervical malignant transformation identifies RFC4 as a novel diagnostic and prognostic biomarker

Jianwei Zhang, et al.

Supplementary Figures 1-10

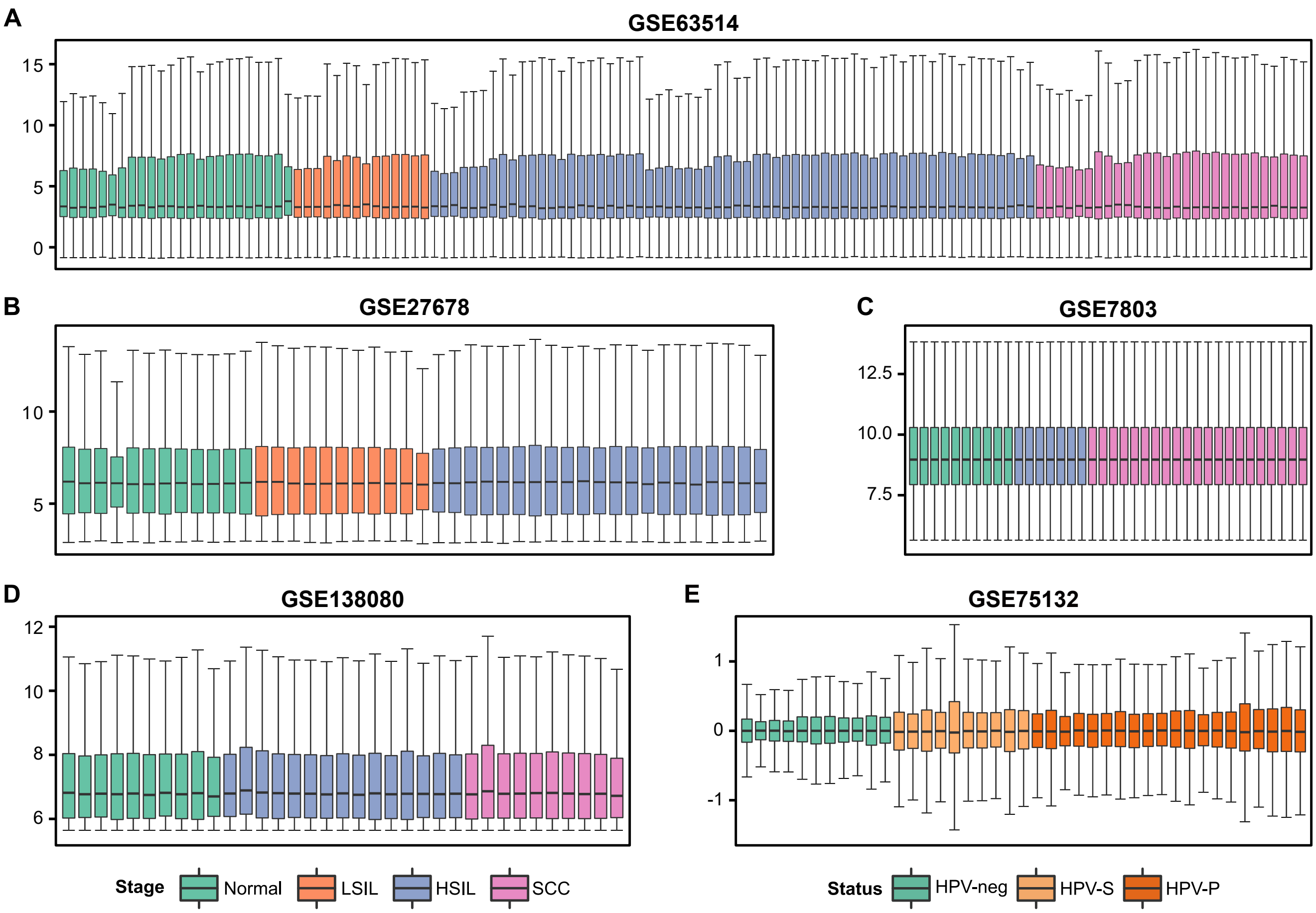

Figure S1. The quality control of selected datasets. (A-E) Boxplots of normalized log2-transformed expression data extracted from series matrix files of GSE63514, GSE27678, GSE7803, GSE138080 and GSE75132, respectively. The x-axis represents the samples, and the y-axis represents expression values. The black line in the box plot represents the median value of gene expression. LSIL/HSIL, low/high-grade squamous intraepithelial lesions; SCC, cervical squamous cell carcinoma; HPV-S, HPV16 persistent infection without progression; HPV-P, HPV16 persistent infection with progression.

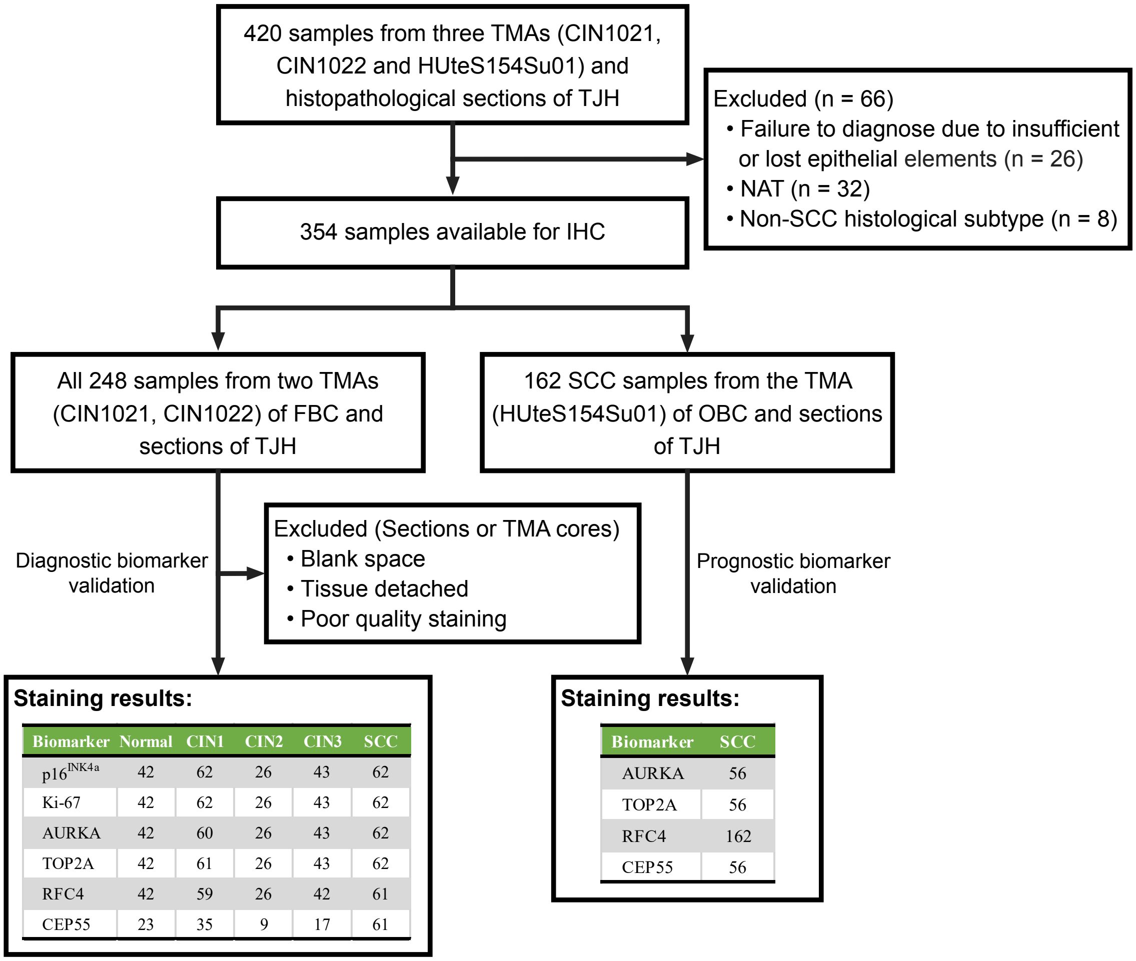

Biomarker	Normal	CIN1	CIN2	CIN3	SCC
p16^{INK4a}	42	62	26	43	62
Ki-67	42	62	26	43	62
AURKA	42	60	26	43	62
TOP2A	42	61	26	43	62
RFC4	42	59	26	42	61
CEP55	23	35	9	17	61

Biomarker	SCC
AURKA	56
TOP2A	56
RFC4	162
CEP55	56

Figure S2. The flow of sample selection and immunohistochemistry (IHC), related to Table S2.

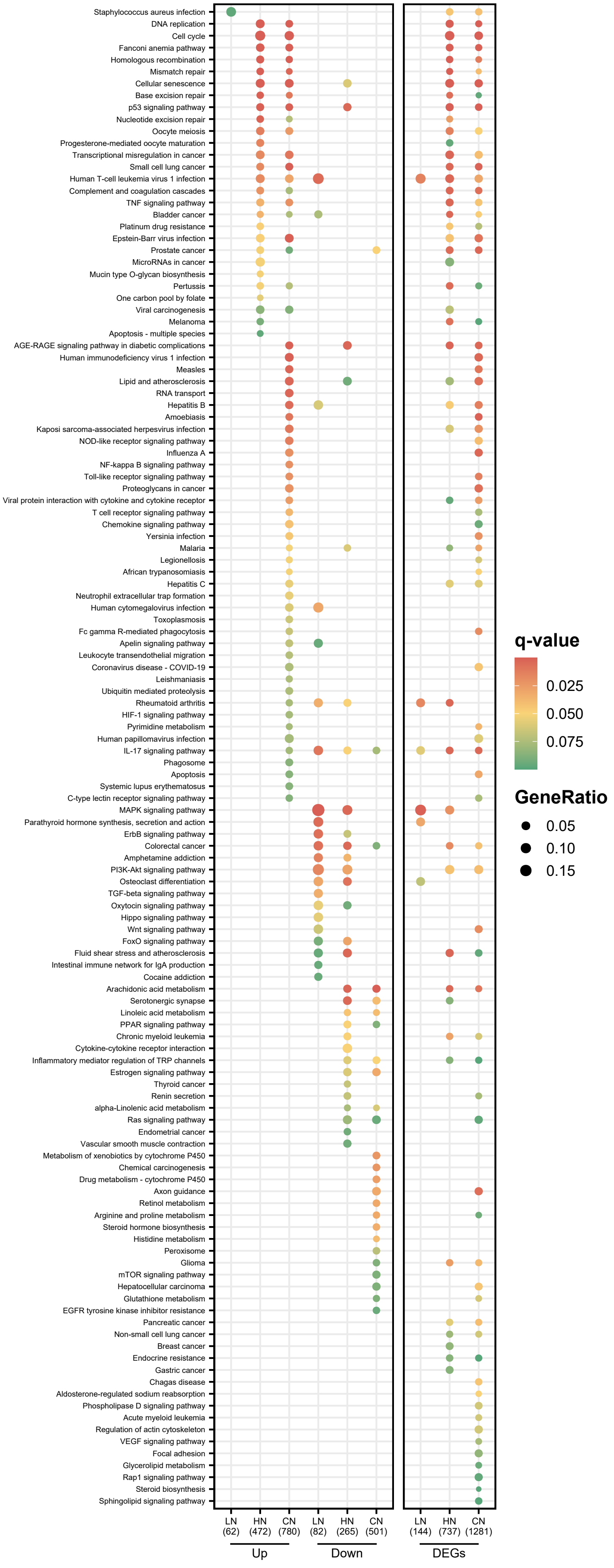

Figure S3. Pathway enrichment and comparison. Dot plot showing all significantly enriched KEGG pathways in up- and downregulated genes (Gene Sets1, left panel) and DEGs (right panel) of each comparison group. Dot color indicates the *q*-value of the enrichment test; dot size represents the fraction of genes annotated to each pathway.

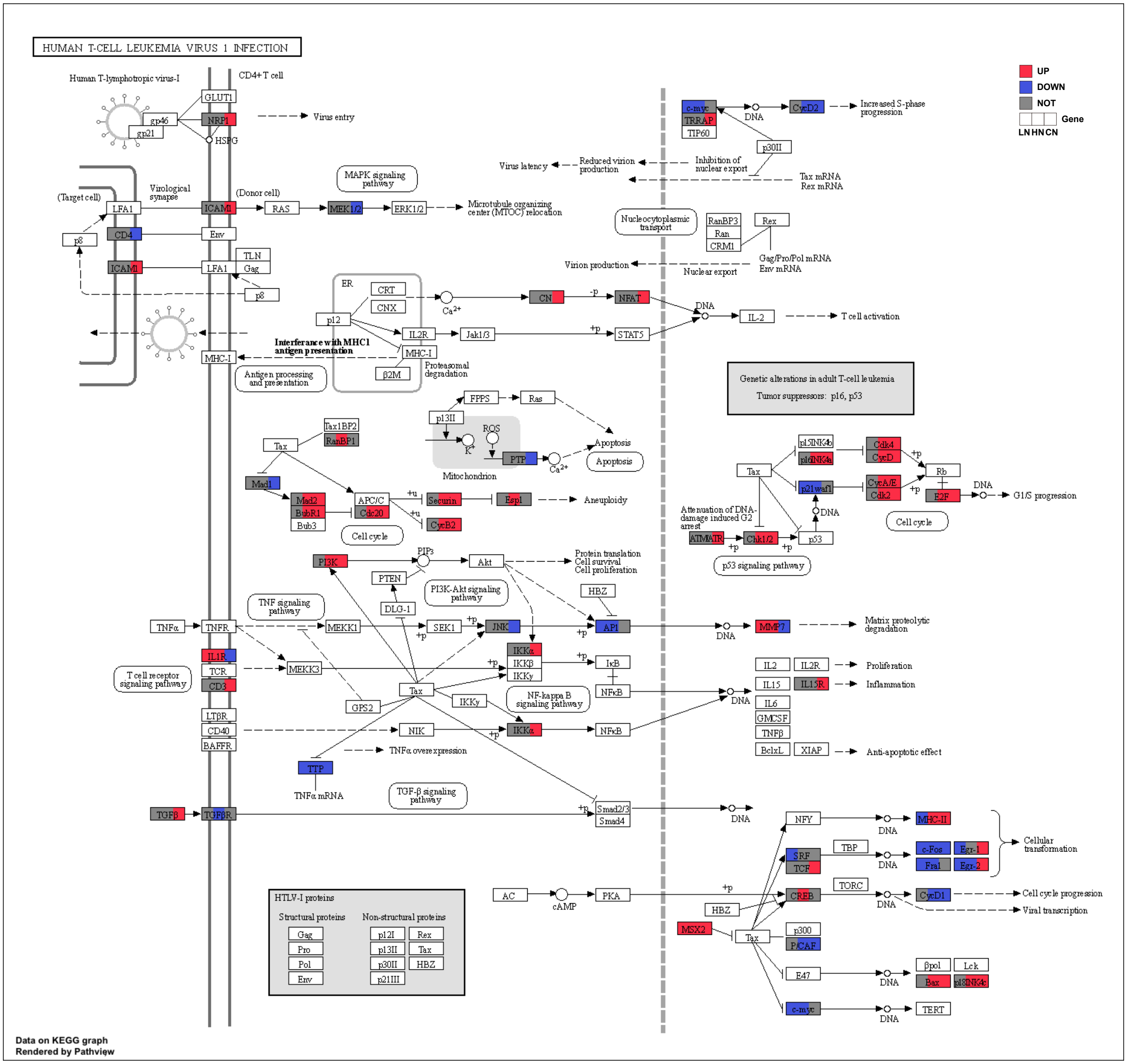

Figure S4. Human T-cell leukemia virus 1 (HTLV-1) infection signaling pathway map. Each box is divided into three sub-boxes equally. The color of each sub-box indicates whether the gene is differentially expressed among LN, HN and CN from left to right. Red indicates upregulation, blue indicates downregulation, and grey indicates not significant. Genes that are differentially expressed in at least one group are colored.

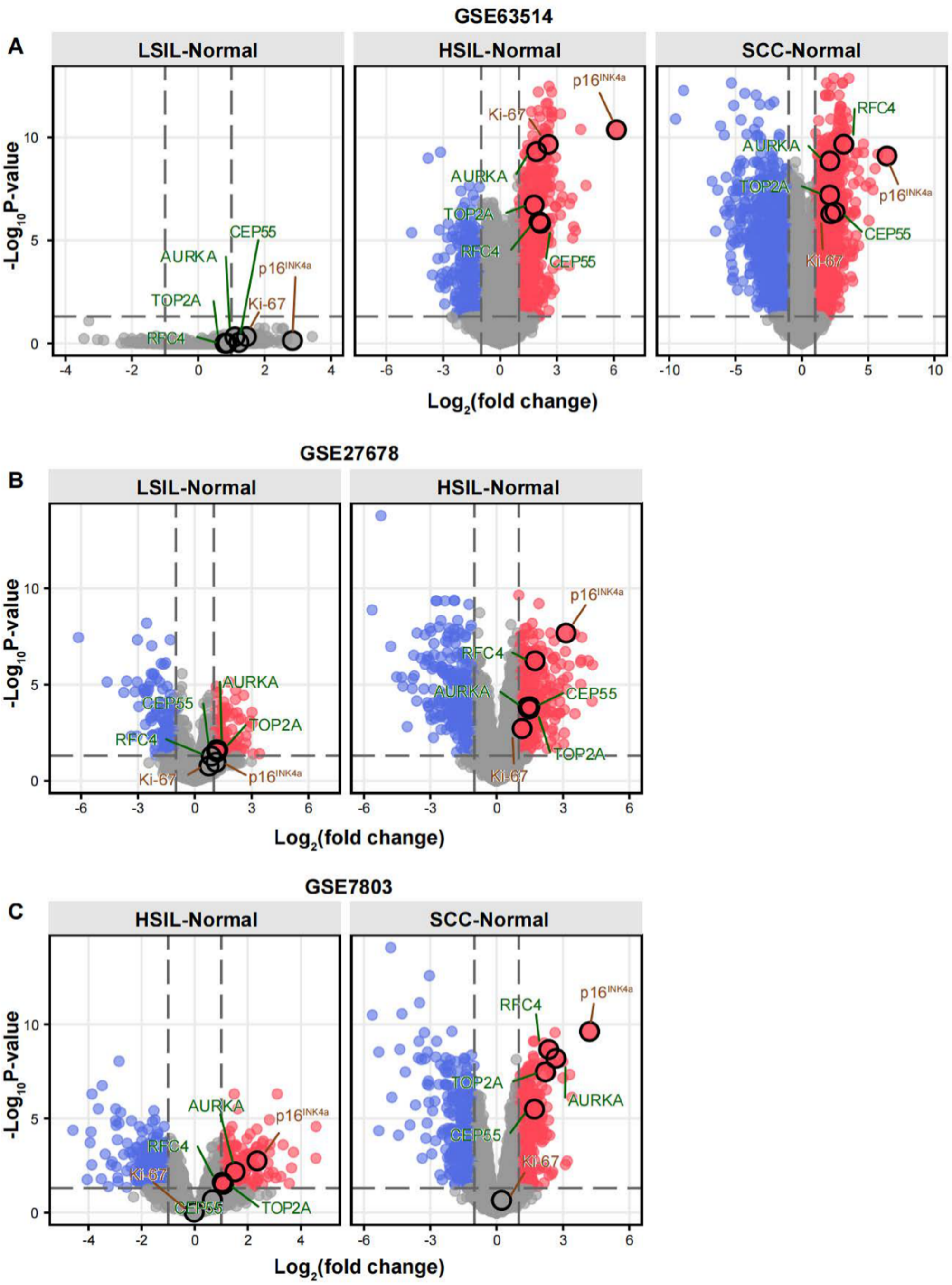

Figure S5. Differential expression of total genes in each comparison group of the discovery datasets. Respective volcano plots of **(A)** GSE63514, **(B)** GSE27678 and **(C)** GSE7803. Red and blue dots represent up- and downregulated genes, respectively; grey dots represent non-statistically significant genes. Vertical dashed lines indicate a 2-fold change cutoff in either direction, and horizontal dashed lines indicate an adjusted *p*-value cutoff of 0.05. $p16^{INK4a}$, Ki-67, and four hub genes are circled and labeled with gene symbols.

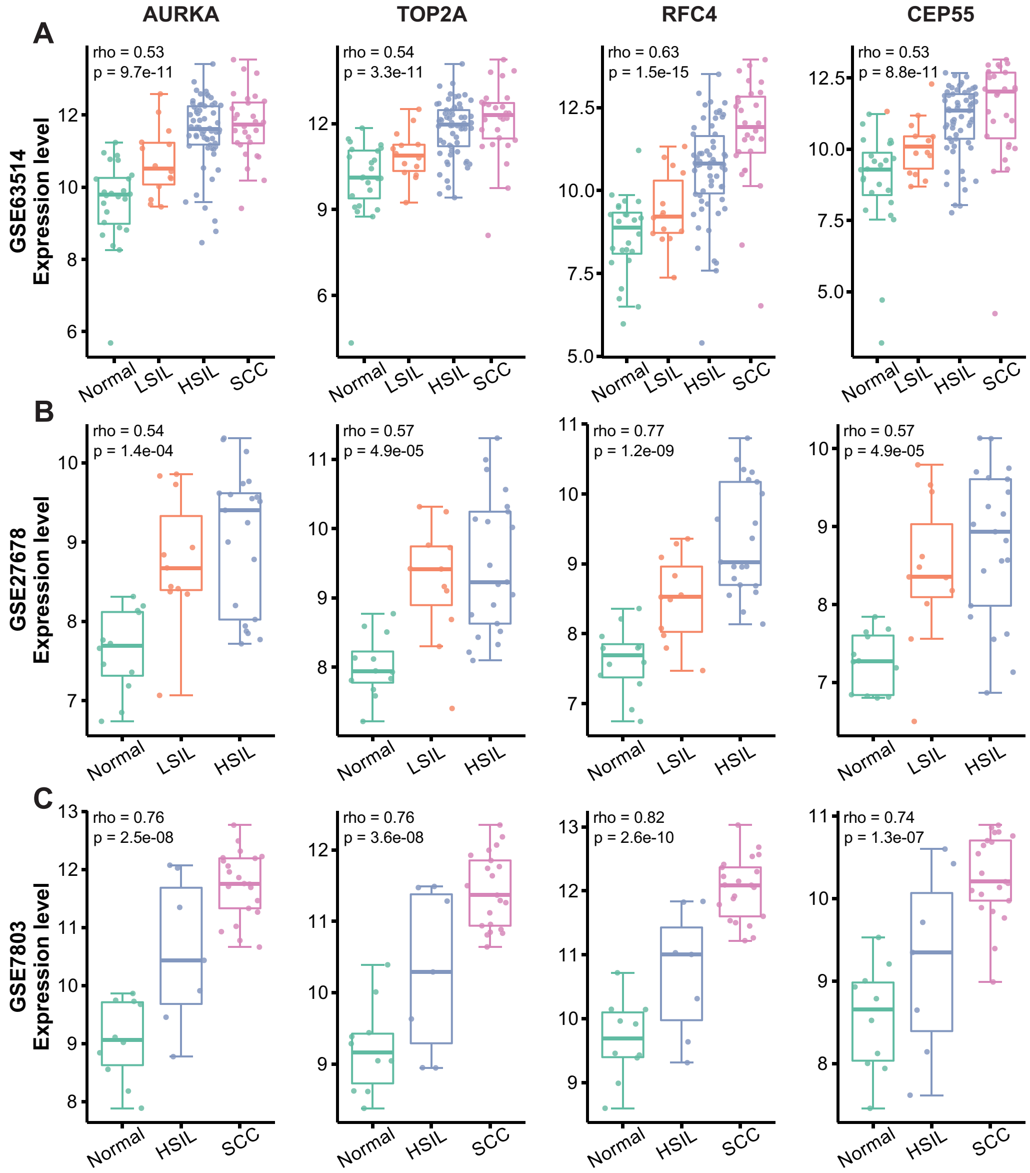

Figure S6. Correlation between hub gene expression and severity of cervical lesion in the discovery datasets. **(A-C)** Boxplots of hub gene expression according to disease stage, with Spearman's rho and *p*-values presented in the upper left corner.

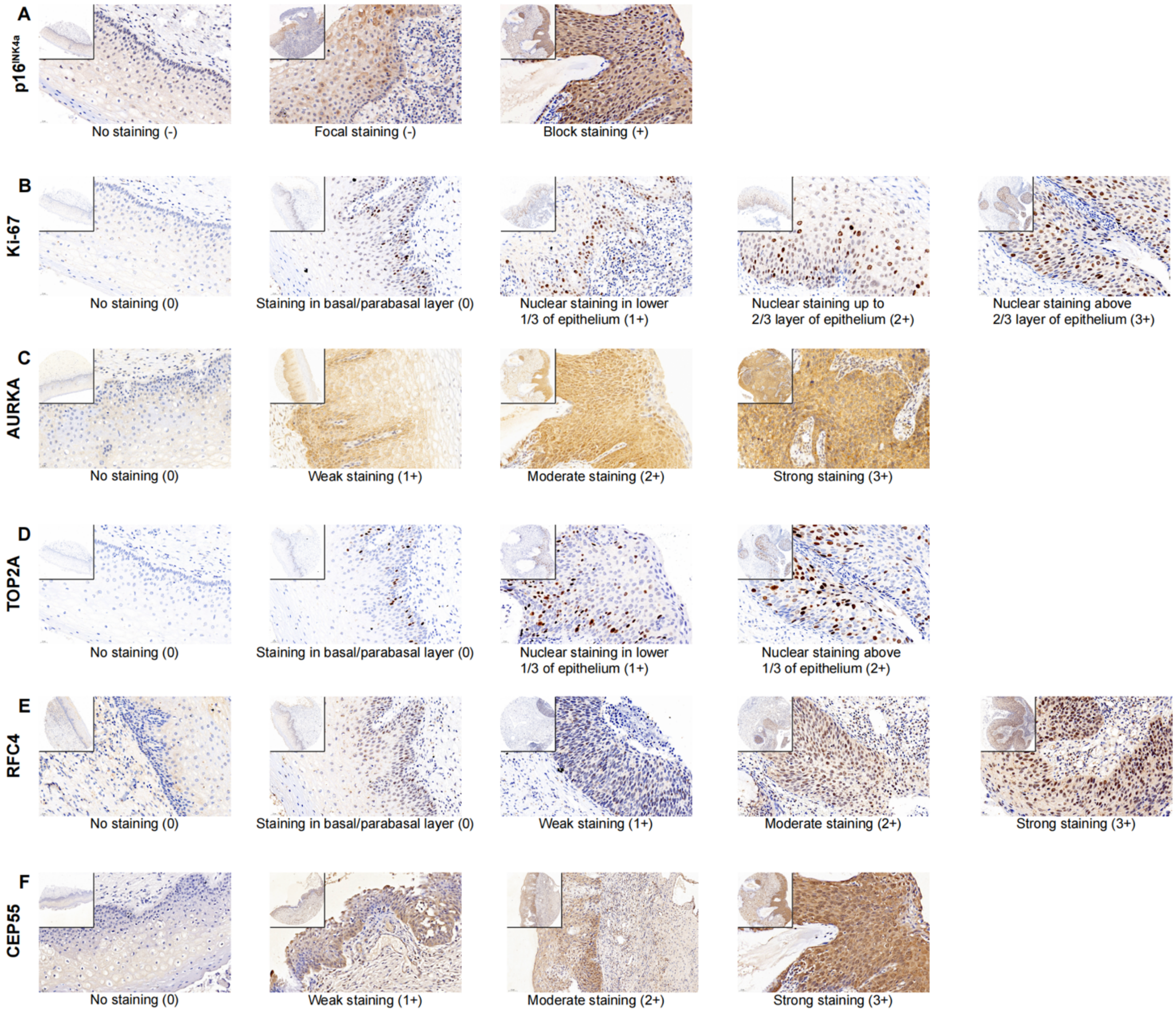

Figure S7. Schematic diagram of immune scoring criteria, related to Table S5. (A-F) Representative IHC staining patterns of p16^{INK4a}, Ki-67, AURKA, TOP2A, RFC4 and CEP55, respectively. Original magnification 400×. Inserts, original magnification 100×.

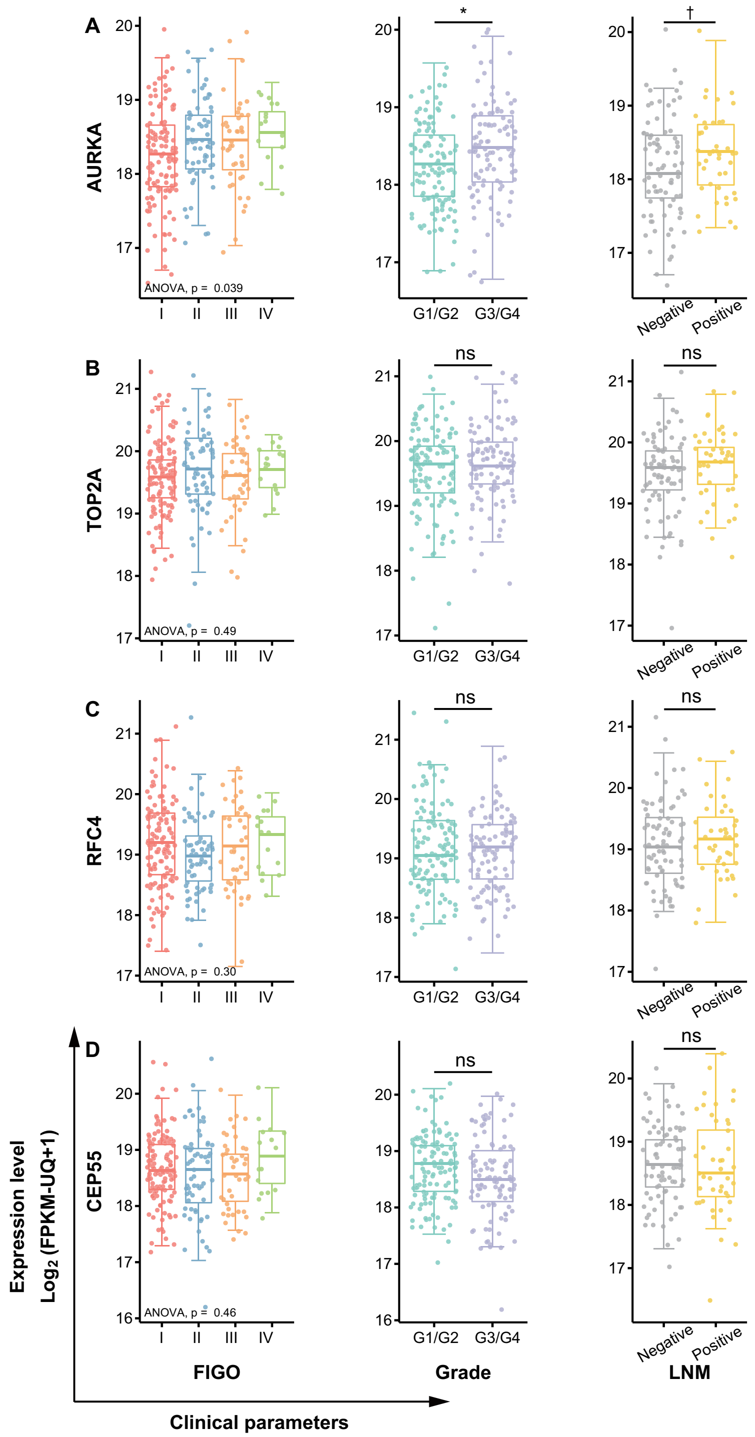

Figure S8. Assessment of the relationship between hub gene expression and clinical parameters in SCC patients from the TCGA cohort. (A-D) Boxplots showing mRNA expression of hub genes stratified by FIGO stage, histologic grade, and lymph node metastasis (LNM). The x-axis represents clinical parameters, and the y-axis represents the gene expression level. One-way ANOVA test and Student's t-test were applied to each comparison accordingly. * $0.01 < p < 0.05$; † $p < 0.1$; ns, $p \geq 0.1$.

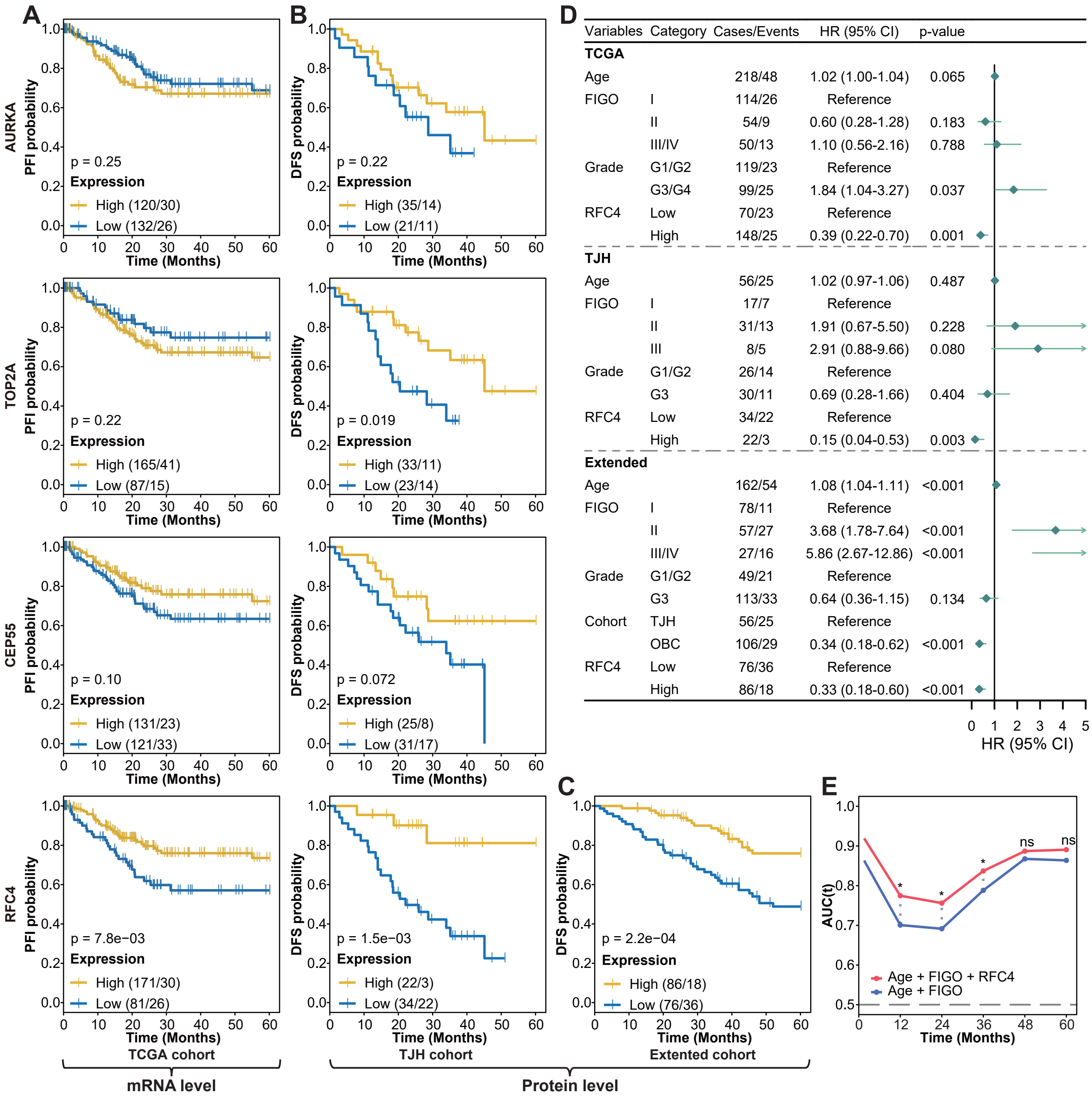

Figure S9. Univariate and multivariate survival analysis for PFI/DFS in SCC patients, related to Fig. 6. Five-year Kaplan-curves for PFI/DFS in SCC patients stratified by the hub gene expression (mRNA and protein) from **(A)** TCGA, **(B)** TJH and **(C)** Extended cohorts. The number of cases and events are shown in the plots. The *p*-values were calculated with the log-rank test. The optimal cutoff values for HSCORE or staining intensity determined by the *surv_cutpoint* function from the survminer package were 140 for AURKA, 1 (staining intensity) for CEP55, 25 for TOP2A, and 115/110 (TJH/Extended) for RFC4. **(D)** Forest plot of multivariate Cox regression with clinical features and RFC4 expression taken into account in three cohorts. The main effects are shown as hazard ratios with 95% confidence intervals. **(E)** Time-dependent AUC for combined RFC4 expression and clinical covariate model (red) and clinical covariate-only model (blue). The significant difference in the AUC was estimated at 1, 2, 3, 4 and 5 years, and adjusted *p*-values were calculated. HR, hazard ratio; CI, confidence interval; AUC, area under the ROC curve. * $0.01 < p < 0.05$; ns, $p \geq 0.1$.

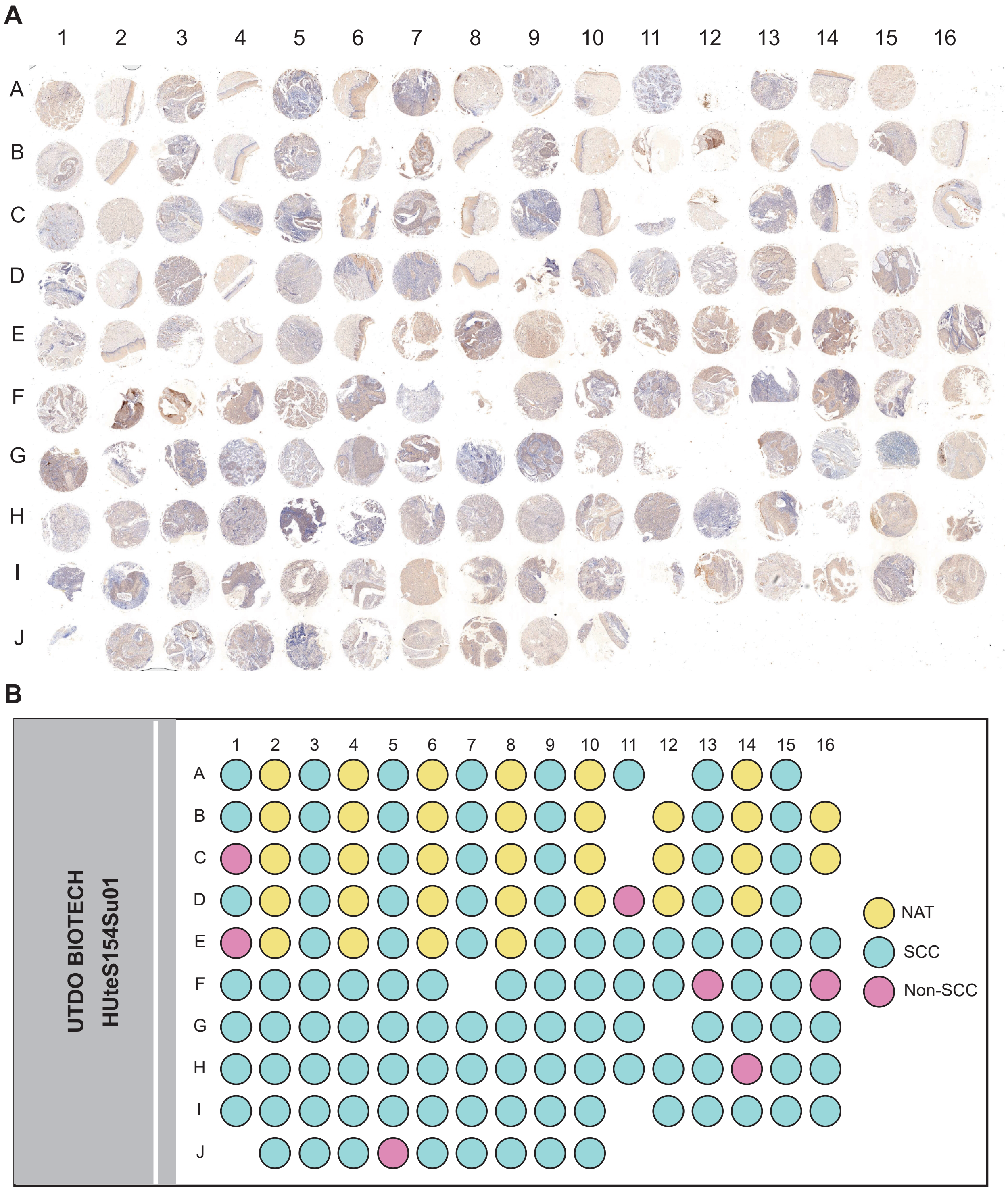

Figure S10. The illustration of the tissue microarray (HUteS154Su01; TMA) from Outdo Biotech. Co., Ltd. **(A)** Scanned high-resolution image of the TMA stained for RFC4. **(B)** Schematic layout of the TMA section. NAT, histologically normal tissue adjacent to the tumor. Non-SCC, non-squamous cell carcinoma of cervix.

Supplementary Tables 1-6, 10-16

Table S1. Microarray datasets analyzed in this study.

Dataset	Year	Platform	Country	Participant				Type
				**Normal[c]**	**LSIL**	**HSIL**	**SCC**	
GSE63514[a]	2015	HG-U133 Plus 2.0	USA	24	14	62	28	Discovery
GSE27678[b]	2013	HG-U133A 2.0	UK	12	11	21	-	Discovery
GSE7803	2007	HG-U133A	USA	10	-	7	21	Discovery
GSE138080	2020	G4112F (Feature Number version)	Netherlands	10	-	15	10	Validation
				**HPV-neg**	**HPV-S**	**HPV-P**		
GSE75132	2016	HG-U133 Plus 2.0	Germany	11	10	20		Validation

[a]GSE63514 contains five types of samples: normal, CIN1, CIN2, CIN3 and SCC. CIN1 was defined as LSIL and CIN2/3 as HSIL.

[b]GSE27678 contains two platforms, and the dataset of HG-U133A 2.0 platform was analyzed.

[c]Of normal tissues, 22 out of 24 and 1 out of 10 samples were HR-HPV positive in GSE63514 and GSE7803 but unknown for GSE27678.

Abbreviations: LSIL/HSIL, low/high-grade squamous intraepithelial lesions; SCC, cervical squamous cell carcinoma; HPV-S, HPV16 persistent infection without progression; HPV-P, HPV16 persistent infection with progression.

Table S2. Histology information of all tissue specimens, related to Fig. S2.

Diagnosis	CIN1021[a]	CIN1022[a]	TJH[b]	HUteS154Su01[c]	Total
Normal or Cervicitis	21	18	3	-	42
CIN1	39	23	-	-	62
CIN2	10	16	-	-	26
CIN3	18	26	3	-	47
Squamous cell carcinoma	5	10	56	106	177
Adenocarcinoma	1	-	-	2	3
Adenosquamous carcinoma	-	-	-	4	4
SCNEC mixed with adenocarcinoma	-	-	-	1	1
NAT	-	-	-	32	32
Failure to diagnose[d]	8	9	-	9	26
**Total**	102	102	62	154	420

[a]Tissue microarrays (TMAs) CIN1021 and CIN1022 were obtained from Fanpu Biotech. Co., Ltd. (FBC).

[b]The collection of histopathological sections obtained from Tongji Hospital was named TJH.

[c]Tissue microarray HUteS154Su01 was obtained from Outdo Biotech. Co., Ltd. (OBC).

[d]Failure to diagnose was attributed to insufficient or lost epithelial elements.

Abbreviations: CIN, cervical intraepithelial neoplasia; SCNEC, small cell neuroendocrine carcinoma; NAT, histologically normal tissue adjacent to the tumor.

Table S3. Summary of DEGs and Gene Sets1&2.

Dataset	LSIL-Normal		HSIL-Normal		SCC-Normal		Stepwise genes[a]	Gene Sets2
	UP	DOWN	UP	DOWN	UP	DOWN		
GSE63514	0	0	744	316	1849	1105	7885	725
GSE27678	113	132	420	296	-	-	6775	123
GSE7803	-	-	220	244	384	357	4636	121
**Gene Sets1**	113	132	1122	626	1971	1256	-	-

[a]Stepwise genes refers to genes gradually increase or decrease in expression with the progression of cervical lesion.

Table S4. Antibodies used for immunohistochemical staining.

Antibody	Antigen retrieval	Dilution	Host species	Vendor (Cat.No.)
Anti-p16^{INK4a}	EDTA pH 9.0	1:100	Rabbit (monoclonal)	Abcam (ab108349)
Anti-Ki-67	EDTA pH 9.0	1:200	Mouse (monoclonal)	Immunoway (YM6189)
Anti-AURKA	EDTA pH 9.0	1:500	Rabbit (polyclonal)	Abcam (ab1287)
Anti-TOP2A	EDTA pH 9.0	1:2000	Rabbit (monoclonal)	Abcam (ab52934)
Anti-RFC4	EDTA pH 9.0	1:500	Mouse (monoclonal)	Abcam (ab156780)
Anti-CEP55	EDTA pH 9.0	1:100	Rabbit (polyclonal)	SinoBiological (200475-T08)

Abbreviations: EDTA, Ethylenediaminetetraacetic acid.

Table S5. Immunohistochemical scoring system for noninvasive squamous epithelia.

Biomarker	Location	IHC score	Criteria	Result
p16^{INK4a}	Nucleus + Cytoplasm		Staining pattern	
		-	No staining or focal/sporadic epithelial staining	Negative
		+	Continuous nuclear staining (diffuse block staining) with or without cytoplasmic reactivity in basal and parabasal layer	Positive
Ki-67	Nucleus		Staining distribution	
		0	No staining or staining in basal/parabasal layer	Negative
		1+	Nuclear staining in lower 1/3 of epithelium	Positive
		2+	Nuclear staining up to 2/3 layer of epithelium	Positive
		3+	Nuclear staining above 2/3 layer of epithelium	Positive
AURKA	Cytoplasm[a]		Staining intensity	
		0	No staining	Negative
		1+	Weak staining	Negative
		2+	Moderate staining	Positive
		3+	Strong staining	Positive
TOP2A	Nucleus		Staining distribution	
		0	No staining or staining in basal/parabasal layer	Negative
		1+	Nuclear staining in lower 1/3 of epithelium	Positive
		2+	Nuclear staining above 1/3 of epithelium	Positive
RFC4	Nucleus + Cytoplasm		Staining pattern	
		0	No staining, staining only in basal/parabasal layer or cytoplasmic-only staining	Negative
		1+	Weak nuclear staining with or without cytoplasmic reactivity	Negative
		2+	Moderate nuclear staining with or without cytoplasmic reactivity	Positive
		3+	Strong nuclear staining with or without cytoplasmic reactivity	Positive
CEP55	Cytoplasm		Staining intensity	
		0	No staining	Negative
		1+	Weak staining	Negative
		2+	Moderate staining	Positive
		3+	Strong staining	Positive

[a]Nuclear and cytoplasmic staining of AURKA was observed in SCC.

Table S6. Clinical characteristics of the collected cohorts for survival analysis.

Characteristics	TCGA (n = 252)	TJH (n = 56)	OBC (n = 106)
Age, years, median (IQR)	47.0 (39.0, 57.2)	50.5 (43.5, 57.0)	45.5 (41.0, 52.0)
FIGO stage, n (%)			
I	125 (49.6)	17 (30.4)	61 (57.5)
II	62 (24.6)	31 (55.4)	26 (24.5)
III	42 (16.7)	8 (14.3)	18 (17.0)
IV	16 (6.3)	0 (0.0)	1 (0.9)
Unknown	7 (2.8)	0 (0.0)	0 (0.0)
Histologic grade, n (%)			
G1	12 (4.8)	2 (3.6)	0 (0.0)
G2	109 (43.3)	24 (42.9)	23 (21.7)
G3	102 (40.5)	30 (53.6)	83 (78.3)
G4	1 (0.4)	0 (0.0)	0 (0.0)
GX	20 (7.9)	0 (0.0)	0 (0.0)
Unknown	8 (3.2)	0 (0.0)	0 (0.0)
Lymph node metastasis, n (%)			
Negative	80 (31.7)	35 (62.5)	88 (83.0)
Positive	43 (17.1)	21 (37.5)	18 (17.0)
Unknown	129 (51.2)	0 (0.0)	0 (0.0)
HPV status, n (%)			
Negative	4 (1.6)	4 (7.1)	14 (13.2)
Positive	141 (56.0)	18 (32.1)	83 (78.3)
Unknown	107 (42.5)	34 (60.7)	9 (8.5)
Follow-up, months, median (95% CI)[a]	27.4 (25.0-33.9)	35.7 (26.5-39.1)	76.0 (73.0-78.0)
Vital status, n (%)			
Alive	192 (76.2)	40 (71.4)	79 (74.5)
Dead	60 (23.8)	16 (28.6)	27 (25.5)
Recurrence, n (%)			
Yes	57 (22.6)	25 (44.6)	31 (29.2)
No	195 (77.4)	31 (55.4)	75 (70.8)

[a]Median follow-up time was calculated using reverse Kaplan-Meier method.

Abbreviations: IQR, interquartile range; FIGO, International Federation of Gynecology and Obstetrics; CI, confidence interval.

Table S10. Summary of the hub gene expression related to cancer progression.

Gene	Type of tumor	Method	Sample size	Expression pattern	Refs
AURKA	Cervical squamous cell carcinoma (CSCC)	IHC	Total samples (n = 131): 20 normal cervix, 35 CIN3, 76 CSCC	Normal < CIN3 = CSCC	1
	Barrett's adenocarcinoma	IHC	Total samples (n = 112): 25 normal esophageal mucosa, 25 GM, 25 IM, 16 LG-IEN, 11 HG-IEN, 10 Bac	Normal < GM < IM < LG-IEN < HG-IEN < BAc (Increased in higher grade)	2
	Colorectal carcinoma (CRC)	IHC	Total samples (n = 60): 40 conventional adenomas, 20 ACIC (ACIC-A + ACIC-E)	Conventional adenoma < ACIC-A < ACIC (Increased in higher grade)	3
	Colorectal carcinoma	qPCR	Total samples (n = 75): 25 normal colon mucosa, 25 CRAP, 25 CRC	Normal < CRAP = CRC	4
TOP2A	Cervical cancer	IHC	Total samples (n = 64): 5 normal cervix, 10 HSIL, 49 cancer	Normal < HSIL = Cancer	5
	Colorectal carcinoma	IHC	Total samples (n = 215): 27 normal mucosa, 72 IBD, 68 adenoma, 48 carcinoma	Normal < IBD < Carcinoma Normal < Adenoma < Carcinoma (Increased in higher grade)	6
	Oral squamous cell carcinoma (OSCC)	IHC	Total samples (n = 58): 20 normal oral mucosa, 16 OED, 22 OSCC	Normal < OED < OSCC (Increased in higher grade)	7
	Barrett's adenocarcinoma	IHC	Total samples (n = 44): 18 NDBO, 13 DBO, 13 Barrett's adenocarcinoma	NDBO < DBO NDBO < Barrett's denocarcinoma	8
RFC4	Cervical squamous cell carcinoma (CSCC)	Microarray + IHC	Microarray samples (n = 38): 10 normal cervix, 7 HSIL, 21CSCC IHC samples (n = 82): 15 normal cervix, 20 LSIL, 21 HSIL, 26 CSCC	IHC: Normal < CSCC HSIL< CSCC	9
CEP55	Head and neck squamous cell carcinoma (HNSCC)	IHC	IHC samples (n = 15): 5 normal oral mucosa, 5 oral dysplasia, 5 HNSCC	Normal < HNSCC < Dysplasia	10
	Colorectal carcinoma	WB + IHC	Total samples (n = 43): 15 noncancerous mucosae, 13 adenoma, 15 CRC	WB: Normal < CRC IHC: Adenoma < CRC	11

Abbreviations: CIN, cervical intraepithelial neoplasia; GM, columnar intestinal metaplasia–negative esophageal mucosa [gastric type]; IM, columnar intestinal metaplasia–positive esophageal mucosa; LG-IEN, low-grade intraepithelial neoplasia; HG-IEN, high-grade intraepithelial neoplasia; BAc, Barrett's esophageal adenocarcinoma; ACIC, adenomas containing invasive carcinoma; ACIC-A, ACIC sectors corresponding to adenoma tissue with low- and high-grade dysplasia; ACIC-E, ACIC sectors corresponding to early invasive carcinoma; CRAP, colorectal adenomatous polyp; HSIL: high-grade squamous intraepithelial lesion; IBD, inflammatory bowel disease; OED, oral epithelial dysplasia; NDBO, non-dysplastic Barrett's esophagus; DBO, dysplastic Barrett's esophagus; LSIL, low-grade squamous intraepithelial lesion; WB, western blot.

References:

1. Twu NF, Yuan CC, Yen MS, Lai CR, Chao KC, Wang PH, Wu HH, Chen YJ: Expression of Aurora kinase A and B in normal and malignant cervical tissue: High Aurora A kinase expression in squamous cervical cancer. European Journal of Obstetrics & Gynecology and Reproductive Biology 2009, 142:57-63.

2. Rugge M, Fassan M, Zaninotto G, Pizzi M, Giacomelli L, Battaglia G, Rizzetto C, Parente P, Ancona E: Aurora kinase A in Barrett's carcinogenesis. Hum Pathol 2010, 41:1380-1386.

3. Casorzo L, Dell'Aglio C, Sarotto I, Risio M: Aurora kinase A gene copy number is associated with the malignant transformation of colorectal adenomas but not with the serrated neoplasia progression. Hum Pathol 2015, 46:411-418.

4. Kasap E, Gerceker E, Boyacıoglu S, Yuceyar H, Yıldırm H, Ayhan S, Korkmaz M: The potential role of the NEK6, AURKA, AURKB, and PAK1 genes in adenomatous colorectal polyps and colorectal adenocarcinoma. Tumour Biol 2016, 37:3071-3080.

5. Davidson B, Goldberg I, Lerner-Geva L, Gotlieb WH, Ben-Baruch G, Novikov I, Kopolovic J: Expression of topoisomerase II and Ki-67 in cervical carcinoma--clinicopathological study using immunohistochemistry. Apmis 2000, 108:209-215.
6. Stromar IK, Jakic-Razumovic J: The value of immunohistochemical determination of topoisomerase IIalpha and Ki67 as markers of cell proliferation and malignant transformation in colonic mucosa. Appl Immunohistochem Mol Morphol 2014, 22:524-529.
7. Shamaa AA, Zyada MM, Wagner M, Awad SS, Osman MM, Abdel Azeem AA: The significance of Epstein Barr virus (EBV) & DNA topoisomerase II alpha (DNA-Topo II alpha) immunoreactivity in normal oral mucosa, oral epithelial dysplasia (OED) and oral squamous cell carcinoma (OSCC). Diagn Pathol 2008, 3:45.
8. Rossi E, Villanacci V, Bassotti G, Donato F, Festa A, Cengia G, Grisanti S, Cestari R: TOPOIIalpha and HER-2/neu overexpression/amplification in Barrett's oesophagus, dysplasia and adenocarcinoma. Histopathology 2010, 57:81-89.
9. Zhai Y, Kuick R, Nan B, Ota I, Weiss SJ, Trimble CL, Fearon ER, Cho KR: Gene expression analysis of preinvasive and invasive cervical squamous cell carcinomas identifies HOXC10 as a key mediator of invasion. Cancer Res 2007, 67:10163-10172.
10. Waseem A, Ali M, Odell EW, Fortune F, Teh MT: Downstream targets of FOXM1: CEP55 and HELLS are cancer progression markers of head and neck squamous cell carcinoma. Oral Oncology 2010, 46:536-542.
11. Sakai M, Shimokawa T, Kobayashi T, Matsushima S, Yamada Y, Nakamura Y, Furukawa Y: Elevated expression of C10orf3 (chromosome 10 open reading frame 3) is involved in the growth of human colon tumor. Oncogene 2006, 25:480-486.

Table S11. The summary of immunohistochemical scoring results.

Biomarker	Normal		CIN1		CIN2		CIN3	
	Total	n (%)	Total	n (%)	Total	n (%)	Total	n (%)
p16^{INK4a}								
-	42	37 (88.1)	62	27 (43.5)	26	2 (7.7)	43	3 (7.0)
+		5 (11.9)		35 (56.5)		24 (92.3)		40 (93.0)
Ki-67								
0	42	40 (95.2)	62	42 (67.7)	26	4 (15.4)	43	2 (4.7)
1+		1 (2.4)		18 (29.0)		5 (19.2)		3 (7.0)
2+		1 (2.4)		2 (3.2)		16 (61.5)		8 (18.6)
3+		0 (0.0)		0 (0.0)		1 (3.8)		30 (69.8)
AURKA								
0	42	21 (50.0)	60	13 (21.7)	26	3 (11.5)	43	3 (7.0)
1+		11 (26.2)		24 (40.0)		1 (3.8)		4 (9.3)
2+		10 (23.8)		20 (33.3)		21 (80.8)		19 (44.2)
3+		0 (0.0)		3 (5.0)		1 (3.8)		17 (39.5)
TOP2A								
0	42	42 (100.0)	61	48 (78.7)	26	6 (23.1)	43	2 (4.7)
1+		0 (0.0)		11 (18.0)		4 (15.4)		1 (2.3)
2+		0 (0.0)		2 (3.3)		16 (61.5)		40 (93.0)
RFC4								
0	42	30 (71.4)	59	32 (54.2)	26	3 (11.5)	42	3 (7.1)
1+		10 (23.8)		19 (32.2)		1 (3.8)		1 (2.4)
2+		2 (4.8)		8 (13.6)		15 (57.7)		19 (45.2)
3+		0 (0.0)		0 (0.0)		7 (26.9)		19 (45.2)
CEP55								
0	23	8 (34.8)	35	5 (14.3)	9	0 (0.0)	17	1 (5.9)
1+		8 (34.8)		10 (28.6)		2 (22.2)		1 (5.9)
2+		7 (30.4)		13 (37.1)		3 (33.3)		11 (64.7)
3+		0 (0.0)		7 (20.0)		4 (44.4)		4 (23.5)

Table S12. Characteristics of studies assessing $p16^{INK4a}$, Ki-67, TOP2A and ProExC immunohistochemically.

Refs	Total	Non-dysplasia n (%)	CIN1 n (%)	CIN2 n (%)	CIN3 n (%)	Cervical cancer n (%)
$p16^{INK4a}$						
Murphy 2005 [1]	147	0/20 (0)	38/38 (100)	33/33 (100)	45/46 (97.8)	10/10 (100)
Ishikawa 2006 [2]	143	0/7 (0)	12/37 (32.4)	32/39 (82.1)	41/44 (93.2)	16/16 (100)
Kim 2015 [3]	149	0/17 (0)	22/31 (71.0)	21/25 (84.0)	41/41 (100)	35/35 (100)
Zhong 2015 [4]	1144	72/329 (21.9)	307/456 (67.3)	172/174 (98.9)	162/163 (99.4)	22/22 (100)
Chaloob 2015 [5]	105	-	9/24 (37.5)	19/28 (67.9)		50/53 (94.3)
Kanthiya 2016 [6]	243	5/53 (9.4)	11/106 (10.4)	48/61 (78.7)		21/23 (91.3)
Ki-67						
Kim 2015 [3]	147	0/17 (0)	15/30 (50.0)	16/25 (64.0)	40/40 (100)	35/35 (100)
Zhong 2015 [4]	1145	101/331 (31.5)	359/456 (78.7)	170/174 (98.7)	160/162 (98.8)	22/22 (100)
Kanthiya 2016 [6]	243	6/53 (11.3)	24/106 (22.6)	46/61 (75.4)		23/23 (100)
Howitt 2013 [7]	139	2/25 (8.0)	26/55 (47.3)	9/11 (81.8)	12/16 (75.0)	27/32 (84.4)
Lim 2015 [8]	103	0/3 (0)	20/48 (41.7)	52/52 (100)		-
Mitildzans 2016 [9]	58	2/10 (20.0)	6/20 (30.0)	14/14 (100)	14/14 (100)	-
TOP2A						
Shi 2007 [10]	62	0/14 (0)	26/34 (76.5)	12/14 (85.7)		-
ProExC (TOP2A and MCM2)						
Shi 2007 [10]	62	0/14 (0)	32/34 (94.1)	11/14 (78.6)		-
Ozaki 2010 [11]	252	1/9 (11.1)	65/123 (52.8)	56/57 (98.2)	49/49 (100)	14/14 (100)
Yang 2013 [12]	166	0/19 (0)	27/29 (93.1)	35/35 (100)	63/63 (100)	20/20 (100)

References:

1. Murphy N, Ring M, Heffron C, King B, Killalea AG, Hughes C, Martin CM, McGuinness E, Sheils O, O'Leary JJ: p16(INK4A), CDC6, and MCM5: predictive biomarkers in cervical preinvasive neoplasia and cervical cancer. Journal of Clinical Pathology 2005, 58:525-534.

2. Ishikawa M, Fujii T, Saito M, Nindl I, Ono A, Kubushiro K, Tsukazaki K, Mukai M, Nozawa S: Overexpression of p16 INK4a as an indicator for human papillomavirus oncogenic activity in cervical squamous neoplasia. Int J Gynecol Cancer 2006, 16:347-353.

3. Kim TH, Han JH, Shin E, Noh JH, Kim HS, Song YS: Clinical Implication of p16, Ki-67, and Proliferating Cell Nuclear Antigen Expression in Cervical Neoplasia: Improvement of Diagnostic Accuracy for High-grade Squamous Intraepithelial Lesion and Prediction of Resection Margin Involvement on Conization Specimen. J Cancer Prev 2015, 20:70-77.

4. Zhong P, Li J, Gu Y, Liu Y, Wang A, Sun Y, Lu L: P16 and Ki-67 expression improves the diagnostic accuracy of cervical lesions but not predict persistent high risk human papillomavirus infection with CIN1. Int J Clin Exp Pathol 2015, 8:2979-2986.

5. Chaloob MK, Hussein AG, Qasim BJ: Correlation of P16 (Ink4a) and CK17 to HPV (16E6+18E6) in Premalignant and Malignant Lesions of Uterine Cervix: A Clinicopathologic Study. Iran J Pathol 2016, 11:377-390.

6. Kanthiya K, Khunnarong J, Tangjitgamol S, Puripat N, Tanvanich S: Expression of the p16 and Ki67 in Cervical Squamous Intraepithelial Lesions and Cancer. Asian Pac J Cancer Prev 2016, 17:3201-3206.

7. Howitt BE, Nucci MR, Drapkin R, Crum CP, Hirsch MS: Stathmin-1 expression as a complement to p16 helps identify high-grade cervical intraepithelial neoplasia with increased specificity. Am J Surg Pathol 2013, 37:89-97.

8. Lim S, Lee MJ, Cho I, Hong R, Lim SC: Efficacy of p16 and Ki-67 immunostaining in the detection of squamous intraepithelial lesions in a high-risk HPV group. Oncol Lett 2016, 11:1447-1452.

9. Mitildzans A, Arechvo A, Rezeberga D, Isajevs S: Expression of p63, p53 and Ki-67 in Patients with Cervical Intraepithelial Neoplasia. Turk Patoloji Derg 2017, 33:9-16.

10. Shi J, Liu H, Wilkerson M, Huang Y, Meschter S, Dupree W, Schuerch C, Lin F: Evaluation of p16INK4a, minichromosome maintenance protein 2, DNA topoisomerase II alpha, ProE(x) C, and p16INK4a/ProE(x) C in cervicat squamous intraepithelial lesions. Human Pathology 2007, 38:1335-1344.

11. Ozaki S, Zen Y, Inoue M: Biomarker expression in cervical intraepithelial neoplasia: potential progression predictive factors for low-grade lesions. Human Pathology 2011, 42:1007-1012.

12. Yang QC, Zhu Y, Liou HB, Zhang XJ, Shen Y, Ji XH: A Cocktail of Mcm2 and Top2a, P16(Ink4a) and Ki-67 as Biomarkers for the Improved Diagnosis of Cervical Intraepithelial Lesion. Polish Journal of Pathology 2013, 64:21-27.

Table S13. Concordance analysis between IHC biomarkers.

Biomarker	Concordance, Unweighted kappa coefficient (95% CI)					
	$p16^{INK4a}$	Ki-67	AURKA	TOP2A	RFC4	CEP55
**All Stages**						
$p16^{INK4a}$	100%, 1.00 (1.00-1.00)					
Ki-67	82.6%, 0.62 (0.51-0.71)	100%, 1.00 (1.00-1.00)				
AURKA	81.1%, 0.57 (0.45-0.68)	82.8%, 0.63 (0.52-0.73)	100%, 1.00 (1.00-1.00)			
TOP2A	82.5%, 0.63 (0.53-0.73)	90.2%, 0.80 (0.72-0.87)	82.8%, 0.64 (0.54-0.74)	100%, 1.00 (1.00-1.00)		
RFC4	80.0%, 0.58 (0.47-0.68)	84.3%, 0.68 (0.57-0.76)	82.2%, 0.63 (0.54-0.74)	86.5%, 0.73 (0.63-0.82)	100%, 1.00 (1.00-1.00)	
CEP55	83.4%, 0.60 (0.44-0.74)	77.2%, 0.48 (0.32-0.62)	83.4%, 0.59 (0.44-0.73)	75.9%, 0.47 (0.33-0.60)	79.6%, 0.55 (0.40-0.68)	100%, 1.00 (1.00-1.00)
**HSIL**						
$p16^{INK4a}$	100%, 1.00 (1.00-1.00)					
Ki-67	92.8%, 0.51 (-0.03-0.85)	100%, 1.00 (1.00-1.00)				
AURKA	88.4%, 0.44 (0.11-0.74)	89.9%, 0.54 (0.19-0.80)	100%, 1.00 (1.00-1.00)			
TOP2A	89.9%, 0.41 (-0.03-0.74)	97.1%, 0.84 (0.55-1.00)	87.0%, 0.45 (0.11-0.72)	100%, 1.00 (1.00-1.00)		
RFC4	86.8%, 0.24 (-0.08-0.57)	82.4%, 0.05 (-0.13-0.36)	80.9%, 0.21 (-0.09-0.50)	82.4%, 0.15 (-0.12-0.45)	100%, 1.00 (1.00-1.00)	
CEP55	84.6%, 0.41 (-0.14-0.83)	84.6%, 0.41 (-0.10-0.86)	84.6%, 0.51 (0.00-0.88)	84.6%, 0.41 (-0.12-0.86)	84.0%, 0.40 (-0.12-0.87)	100%, 1.00 (1.00-1.00)

Table S14. Sensitivity, specificity, PPV, NPV, and AUC of six IHC biomarkers for detecting HSIL and HSIL+.

Biomarker	Sensitivity (95% CI), %	Specificity (95% CI), %	PPV (95% CI), %	NPV (95% CI), %	AUC (95% CI)
**HSIL**					
$p16^{INK4a}$	92.8 (83.9-97.6)	61.5 (51.5-70.9)	61.5 (51.5-70.9)	92.8 (83.9-97.6)	0.77 (0.72-0.83)
Ki-67	91.3 (82.0-96.7)	78.8 (69.7-86.2)	74.1 (63.5-83.0)	93.2 (85.7-97.5)	0.85 (0.80-0.90)
AURKA	84.1 (73.3-91.8)	67.6 (57.7-76.6)	63.7 (53.0-73.6)	86.2 (76.7-92.9)	0.76 (0.70-0.82)
TOP2A	88.4 (78.4-94.9)	87.4 (79.4-93.1)	82.4 (71.8-90.3)	91.8 (84.5-96.4)	0.88 (0.83-0.93)
RFC4	88.2 (78.1-94.8)	90.1 (82.5-95.1)	85.7 (75.3-92.9)	91.9 (84.7-96.4)	0.89 (0.84-0.94)
CEP55	84.6 (65.1-95.6)	53.4 (39.9-66.7)	44.9 (30.7-59.8)	88.6 (73.3-96.8)	0.69 (0.59-0.79)
**HSIL+**					
$p16^{INK4a}$	95.4 (90.3-98.3)	61.5 (51.5-70.9)	75.8 (68.5-82.1)	91.4 (82.3-96.8)	0.78 (0.73-0.84)
Ki-67	93.1 (87.4-96.8)	78.8 (69.7-86.2)	84.7 (77.8-90.2)	90.1 (82.1-95.4)	0.86 (0.81-0.90)
AURKA	91.6 (85.5-95.7)	67.6 (57.7-76.6)	78.4 (71.1-84.7)	86.2 (76.7-92.9)	0.80 (0.74-0.85)
TOP2A	91.6 (85.5-95.7)	87.4 (79.4-93.1)	90.2 (83.9-94.7)	89.1 (81.3-94.4)	0.89 (0.85-0.93)
RFC4	91.5 (85.3-95.7)	90.1 (82.5-95.1)	92.2 (86.1-96.2)	89.2 (81.5-94.5)	0.91 (0.87-0.95)
CEP55	89.7 (81.3-95.2)	53.4 (39.9-66.7)	74.3 (64.8-82.3)	77.5 (61.5-89.2)	0.72 (0.64-0.79)

Table S15. Comparison of positive rates of single and combined IHC biomarkers in different cervical lesions (based on sections with p16^{INK4a}, Ki-67, TOP2A and RFC4 evaluated simultaneously).

Biomarker	Results	Normal (n = 42)	CIN1 (n = 59)	CIN2 (n = 26)	CIN3 (n = 42)	SCC (n = 61)
p16^{INK4a}, n (%)	Negative	37 (88.1)	27 (45.8)	2 (7.7)	3 (7.1)	1 (1.6)
	Positive	5 (11.9)	32 (54.2)	24 (92.3)	39 (92.9)	60 (98.4)
Ki-67, n (%)	Negative	40 (95.2)	41 (69.5)	4 (15.4)	2 (4.8)	3 (4.9)
	Positive	2 (4.8)	18 (30.5)	22 (84.6)	40 (95.2)	58 (95.1)
p16^{INK4a} + Ki-67[a], n (%)	Negative	42 (100)	46 (78.0)	4 (15.4)	4 (9.5)	4 (6.6)
	Positive	0 (0)	13 (22.0)	22 (84.6)	38 (90.5)	57 (93.4)
TOP2A, n (%)	Negative	42 (100)	46 (78.0)	6 (23.1)	2 (4.8)	3 (4.9)
	Positive	0 (0)	13 (22.0)	20 (76.9)	40 (95.2)	58 (95.1)
RFC4, n (%)	Negative	40 (95.2)	51 (86.4)	4 (15.4)	4 (9.5)	3 (4.9)
	Positive	2 (4.8)	8 (13.6)	22 (84.6)	38 (90.5)	58 (95.1)

[a]Both p16^{INK4a} and Ki-67 positive.

Table S16. Diagnostic performance of serial and parallel interpretation of IHC biomarker combinations for detecting HSIL and HSIL+ compared to TOP2A and RFC4 alone.

a. HSIL

Biomarker	Sensitivity (95% CI), %	*p*-value[a]	Specificity (95% CI), %	*p*-value[a]	PPV (95% CI), %	NPV (95% CI), %	AUC (95% CI)
**Single-Refs**							
TOP2A	88.2 (78.1-94.8)	Ref/1.000	87.1 (79.0-93.0)	Ref/0.579	82.2 (71.5-90.2)	91.7 (84.2-96.3)	0.88 (0.83-0.93)
RFC4	88.2 (78.1-94.8)	1.000/Ref	90.1 (82.5-95.1)	0.579/Ref	85.7 (75.3-92.9)	91.9 (84.7-96.4)	0.89 (0.84-0.94)
**Serial**							
$p16^{INK4A}$ + Ki-67	88.2 (78.1-94.8)	1.000/1.000	87.1 (79.0-93.0)	1.000/0.579	82.2 (71.5-90.2)	91.7 (84.2-96.3)	0.88 (0.83-0.93)
$p16^{INK4A}$ + TOP2A	85.3 (74.6-92.7)	0.480/0.773	89.1 (81.3-94.4)	0.480/1.000	84.1 (73.3-91.8)	90.0 (82.4-95.1)	0.87 (0.82-0.92)
$p16^{INK4A}$ + RFC4	83.8 (72.9-91.6)	0.546/0.248	93.1 (86.2-97.2)	0.114/0.248	89.1 (78.8-95.5)	89.5 (82.0-94.7)	0.88 (0.83-0.94)
Ki-67 + TOP2A	88.2 (78.1-94.8)	NA[b]/1.000	90.1 (82.5-95.1)	0.248/1.000	85.7 (75.3-92.9)	91.9 (84.7-96.4)	0.89 (0.84-0.94)
Ki-67 + RFC4	80.9 (69.5-89.4)	0.131/0.074	94.1 (87.5-97.8)	0.046/0.134	90.2 (79.8-96.3)	88.0 (80.3-93.4)	0.87 (0.82-0.93)
TOP2A + RFC4	79.4 (67.9-88.3)	0.041/0.041	95.0 (88.8-98.4)	0.013/0.074	91.5 (81.3-97.2)	87.3 (79.6-92.9)	0.87 (0.82-0.93)
**Parallel**							
$p16^{INK4A}$ + Ki-67	95.6 (87.6-99.1)	0.074/0.182	56.4 (46.2-66.3)	<0.001/<0.001	59.6 (49.8-68.9)	95.0 (86.1-99.0)	0.76 (0.71-0.81)
$p16^{INK4A}$ + TOP2A	95.6 (87.6-99.1)	0.074/0.182	61.4 (51.2-70.9)	<0.001/<0.001	62.5 (52.5-71.8)	95.4 (87.1-99.0)	0.78 (0.73-0.84)
$p16^{INK4A}$ + RFC4	97.1 (89.8-99.6)	0.077/0.041	60.4 (50.2-70.0)	<0.001/<0.001	62.3 (52.3-71.5)	96.8 (89.0-99.6)	0.79 (0.74-0.84)
Ki-67 + TOP2A	91.2 (81.8-96.7)	0.480/0.773	77.2 (67.8-85.0)	0.004/0.009	72.9 (62.2-82.0)	92.9 (85.1-97.3)	0.84 (0.79-0.90)
Ki-67 + RFC4	98.5 (92.1-100.0)	0.023/0.023	76.2 (66.7-84.1)	0.015/<0.001	73.6 (63.3-82.3)	98.7 (93.1-100.0)	0.87 (0.83-0.92)
TOP2A + RFC4	97.1 (89.8-99.6)	0.041/0.041	82.2 (73.3-89.1)	0.074/0.013	78.6 (68.3-86.8)	97.6 (91.8-99.7)	0.90 (0.85-0.94)

[a]Exact McNemar's test comparing to TOP2A and RFC4.

[b]No discordant pairs present.

b. HSIL+

Biomarker	Sensitivity (95% CI), %	*p*-value[a]	Specificity (95% CI), %	*p*-value[a]	PPV (95% CI), %	NPV (95% CI), %	AUC (95% CI)
**Single-Refs**							
TOP2A	91.5 (85.3-95.7)	Ref/1.000	87.1 (79.0-93.0)	Ref/0.579	90.1 (83.6-94.6)	88.9 (81.0-94.3)	0.89 (0.85-0.93)
RFC4	91.5 (85.3-95.7)	1.000/Ref	90.1 (82.5-95.1)	0.579/Ref	92.2 (86.1-96.2)	89.2 (81.5-94.5)	0.91 (0.87-0.95)
**Serial**							
$p16^{INK4A}$ + Ki-67	90.7 (84.3-95.1)	1.000/1.000	87.1 (79.0-93.0)	1.000/0.579	90.0 (83.5-94.6)	88.0 (80.0-93.6)	0.89 (0.85-0.93)
$p16^{INK4A}$ + TOP2A	89.1 (82.5-93.9)	0.248/0.646	89.1 (81.3-94.4)	0.480/1.000	91.3 (84.9-95.6)	86.5 (78.4-92.4)	0.89 (0.85-0.93)
$p16^{INK4A}$ + RFC4	88.4 (81.5-93.3)	0.480/0.134	93.1 (86.2-97.2)	0.114/0.248	94.2 (88.4-97.6)	86.2 (78.3-92.1)	0.91 (0.87-0.94)
Ki-67 + TOP2A	89.1 (82.5-93.9)	0.248/0.663	90.1 (82.5-95.1)	0.248/1.000	92.0 (85.8-96.1)	86.7 (78.6-92.5)	0.90 (0.86-0.94)
Ki-67 + RFC4	85.3 (78.0-90.9)	0.080/0.013	94.1 (87.5-97.8)	0.046/0.134	94.8 (89.1-98.1)	83.3 (75.2-89.7)	0.90 (0.86-0.94)
TOP2A + RFC4	84.5 (77.1-90.3)	0.008/0.008	95.0 (88.8-98.4)	0.013/0.074	95.6 (90.1-98.6)	82.8 (74.6-89.1)	0.90 (0.86-0.94)
**Parallel**							
$p16^{INK4A}$ + Ki-67	97.7 (93.4-99.5)	0.013/0.043	56.4 (46.2-66.3)	<0.001/<0.001	74.1 (66.9-80.5)	95.0 (86.1-99.0)	0.77 (0.72-0.82)
$p16^{INK4A}$ + TOP2A	97.7 (93.4-99.5)	0.013/0.043	61.4 (51.2-70.9)	<0.001/<0.001	76.4 (69.1-82.6)	95.4 (87.1-99.0)	0.80 (0.75-0.84)
$p16^{INK4A}$ + RFC4	98.4 (94.5-99.8)	0.016/0.008	60.4 (50.2-70.0)	<0.001/<0.001	76.0 (68.8-82.3)	96.8 (89.0-99.6)	0.79 (0.75-0.84)
Ki-67 + TOP2A	95.3 (90.2-98.3)	0.074/0.302	77.2 (67.8-85.0)	0.004/0.009	84.2 (77.3-89.7)	92.9 (85.1-97.3)	0.86 (0.82-0.91)
Ki-67 + RFC4	99.2 (95.8-100.0)	0.004/0.004	76.2 (66.7-84.1)	0.015/<0.001	84.2 (77.4-89.6)	98.7 (93.1-100.0)	0.88 (0.83-0.92)
TOP2A + RFC4	98.4 (94.5-99.8)	0.008/0.008	82.2 (73.3-89.1)	0.074/0.013	87.6 (81.1-92.5)	97.6 (91.8-99.7)	0.90 (0.86-0.94)

[a]Exact McNemar's test comparing to TOP2A and RFC4.
